# Data‐Driven and Physics‐Informed Modeling Perspectives for Soft Manipulators: From SINDy to Hybrid Intelligence

**DOI:** 10.1002/advs.78334

**Published:** 2026-10-18

**Authors:** Wenbo Hui, Yunyi Wang, Teng Zhang, Yang Chen, Min Pan

**Affiliations:** ^1^ Department of Mechanical Engineering University of Bath Bath UK

**Keywords:** data‐driven modeling, hybrid intelligence, physics‐informed modeling, SINDy, soft manipulators

## Abstract

Soft manipulators offer a route to safer, more adaptive and physically intelligent robots, but their compliant bodies remain difficult to model, predict and control. Large deformation, distributed actuation, hysteresis, material variability and contact‐rich interaction challenge both classical mechanics‐based models and purely data‐driven approaches. Here, we examine emerging modeling perspectives for soft manipulators through a conceptual progression from sparse identification of nonlinear dynamics to hybrid intelligence. We discuss how sparse identification of nonlinear dynamics (SINDy), Koopman learning, neural ordinary differential equations, physics‐informed neural networks, and hybrid physics machine learning models contribute complementary capabilities, including interpretable reduced‐order dynamics, control‐ready prediction, continuous‐time latent modeling, physics‐constrained field reconstruction, and residual correction. We present that no single modeling approach is sufficient for the next generation of soft robotic systems. Instead, progress requires integrated modeling ecosystems that combine mechanical structure, data‐driven discovery and task‐aware adaptation. Such hybrid‐intelligence frameworks can transform models from passive descriptions of deformation into active components of sensing, control, design and decision‐making, enabling soft manipulators that are more interpretable, robust and deployable in real‐world environments.

## Graphical abstract

This Perspective charts the modeling landscape for soft manipulators, spanning sparse system identification, neural dynamics, physics‐informed models and hybrid intelligence. We examine how physics, data, sensing, adaptation and control can assume complementary roles within this landscape, and provide a framework for selecting modeling approaches according to their accuracy, interpretability, computational cost, robustness, deployment constraints and operating regime.

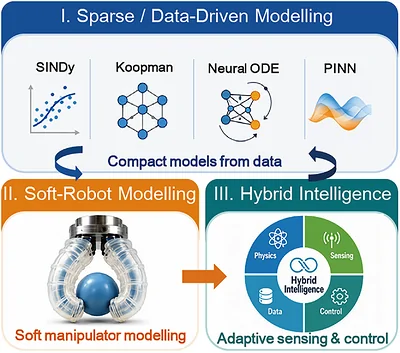

## 1 Introduction: The Soft Manipulator Challenge

Soft manipulators are reshaping the imagination of robotics. Unlike conventional rigid‐link machines, they derive function from compliance, distributed deformation, and intimate physical interaction with the world [1, 2, 3, 4]. Their promise lies not merely in being softer, but in enabling a different class of robotic behavior: safer contact with humans, richer adaptation to uncertainty, and forms of embodied intelligence that emerge from the coupling of material, geometry, actuation, and environment [2, 5, 6, 7, 8]. Yet this promise comes with a profound modeling challenge. The very properties that make soft manipulators powerful: their continuum nature, nonlinear mechanics, high‐dimensional deformations, and strong environmental dependence, also challenge the assumptions that have underpinned much of classical robotics [9, 10, 11, 12, 13]. In soft robotics, modeling is therefore not just a technical subproblem. It is the intellectual bottleneck that determines whether soft machines remain compelling laboratory demonstrations or mature into predictable, controllable, and widely deployable technologies [14, 15, 16].

For much of the field's development, this bottleneck has been approached primarily through physics‐based reasoning. Continuum mechanics, beam formulations, Cosserat rod theory, and finite element methods have provided the core language for describing deformation, force transmission, and constitutive behavior in soft manipulators [9, 10, 17, 18, 19, 20, 21]. These methods have been indispensable: they have grounded the field in mechanics, enabled physically interpretable simulation, and supplied the foundations for design and analysis [15, 20, 22]. But as soft robotic systems become more ambitious, more dexterous, more sensorised, more adaptive, and more embedded in real‐world environments, the limits of relying solely on physics‐based descriptions are becoming increasingly apparent [14, 16, 23]. High‐fidelity models are often too slow for real‐time use, too parameter‐sensitive for scalable deployment, or too fragile under contact‐rich, uncertain, and evolving operating conditions [16, 23, 24, 25]. What the field now confronts is not a failure of mechanics, but the recognition that mechanics alone is no longer sufficient. At the same time, the rise of data‐driven modeling has changed the landscape. Learning‐based methods have opened the possibility of extracting dynamical structure directly from observations, compressing complex behavior into reduced representations, and adapting models online as systems and environments change [26, 27, 28, 29, 30]. For soft manipulators, this shift is especially consequential because so much of their behavior is difficult to derive cleanly from first principles alone: fabrication variability, material hysteresis, fluidic coupling, frictional contact, and unstructured interaction all introduce phenomena that are often only partially modeled or entirely unmodeled [11, 29, 31, 32]. Data‐driven modeling is therefore not merely a convenient alternative; it directly addresses the epistemic limits of purely mechanistic descriptions in embodied, compliant systems [28, 31, 33].

Within this broader shift, sparse identification of nonlinear dynamics (SINDy) offers a particularly revealing conceptual milestone [34, 35, 36, 37]. SINDy captures a deeper ambition emerging across the field: to learn from data without giving up structure, and to discover compact governing dynamics without surrendering interpretability. In this sense, SINDy stands as more than one algorithm among many. It symbolises a transition from modeling as manual derivation to modeling as structured discovery [35, 36]. For soft manipulators, where trust, insight, and control relevance are as important as predictive fit, this transition is especially significant. It suggests that the future of modeling and design need not be split between first‐principles rigidity and black‐box flexibility but may instead lie in frameworks that are both learned and legible [15, 36, 38]. This transition, however, does not end with sparse discovery. Beyond SINDy, the field is rapidly expanding toward operator‐theoretic approaches, neural ordinary differential equations, neural surrogates, latent‐state representations, and other machine‐learning models capable of representing strongly nonlinear, history‐dependent, and partially observed dynamics [27, 30, 39, 40, 41, 42]. These approaches bring expressive power and adaptability, but they also sharpen an important tension. A model that predicts well is not necessarily a model that explains, generalises, or deserves to be trusted in physically consequential settings [30, 43, 44, 45]. In soft robotics, this tension is particularly acute because models are not used in isolation: they sit inside controllers, digital twins, design loops, virtual sensing pipelines, and human‐facing systems [16, 24, 46]. The question is therefore no longer whether learning should be incorporated into soft manipulator modeling. It already has. The real question is what kind of learning the field should build around.

Physics‐informed approaches acknowledge that in soft robotics, learning is most powerful not when it replaces mechanics, but when it is disciplined by it [41, 47, 48, 49, 50]. By embedding conservation laws, constitutive priors, geometric constraints, reduced‐order structure, or simulation‐based supervision into trainable models, these methods seek a more productive balance between flexibility and fidelity [47, 49, 50, 51, 52]. Their significance extends beyond any individual implementation. They represent a philosophical reorientation: from choosing between physics and data, to designing principled interactions between them. For soft manipulators, this reorientation is timely. The field increasingly needs models that are not merely accurate in retrospect but also adaptive in deployment, interpretable in operation, and trustworthy under distribution shifts, contact uncertainty, and changing morphology [14, 15, 16, 45, 50]. From this viewpoint, the trajectory named in this Perspective, from SINDy to hybrid intelligence, should be read as a statement about the evolution of the field itself. It marks a move away from the search for a single best modeling paradigm and toward the recognition that soft manipulation demands a hierarchy of models, each serving different roles across design, control, adaptation, and understanding [24, 38, 46, 53]. A reduced‐order sparse model may be ideal for interpretable control; Physics‐Informed Neural Networks (PINNs) or operator learners may provide adaptable surrogacy; a Cosserat or Finite Element Method (FEM) model may remain essential for mechanistic grounding and validation; a residual learner may capture what the physics misses [20, 35, 41, 49, 54]. The future, in other words, is unlikely to belong to any one of these approaches alone. It will belong to the robot modeling and design ecosystems, where they interact intelligently.

In this Perspective, we use *hybrid intelligence* in a specific system‐level sense. We define *hybrid intelligence* as a modeling architecture that coordinates physics‐based models, learned components, state estimation, adaptation, and task‐level decision‐making within explicit computational and safety constraints. This definition builds on hybrid modeling, where first‐principles and data‐based models are combined within a joint architecture [55], but extends the concept from model construction to the operational coordination of sensing, updating, and action.

The physics‐based component retains geometry, mechanics, and safety‐relevant structure, while the learned component represents residual dynamics, uncertain parameters, latent states, or fast surrogates. Measurements are used to update the state, parameters, and learned correction, and the updated model is then used for control, planning, sensing, and design. Recent robotic architectures have demonstrated these mechanisms through real‐time physical–virtual synchronization, online model refinement, and closed‐loop decision‐making [56, 57, 58].

Accordingly, the simple combination of a physical model and a neural network is not sufficient to establish a hybrid‐intelligence framework. In this Perspective, we propose four qualification criteria. First, the framework should retain explicit physical structure. Second, the learned component should have a clearly defined role associated with model uncertainty, unresolved dynamics, and computational cost. Third, sensory or task feedback should update the state, parameters, learned correction, and model selection. Fourth, the combined framework should directly support task‐level decisions under stated computational, uncertainty, and safety limits [57, 58, 59]. Hybrid physics–machine learning is therefore treated as a model‐level building block, while hybrid intelligence describes the broader architecture that coordinates modeling, inference, adaptation, and action.

## 2 Emerging modeling Paradigms

### 2.1 Interpretable Dynamics via Equation Discovery

Brunton et al. [34] introduced Sparse Identification of Nonlinear Dynamics (SINDy) for discovering governing equations from measurements while retaining an explicit analytical form. Its central assumption is that, although the observed dynamics may be nonlinear and high‐dimensional, the underlying vector field is often governed by only a small number of active terms drawn from a much larger candidate library. As illustrated in Figure 1A, the method starts from measured state trajectories, builds a candidate function library, and then identifies only the small subset of active terms needed to recover the governing dynamics. This sparse structure allows compact models to be identified directly from data, without specifying constitutive relations or reduced‐order equations in advance. Consider a nonlinear dynamical system

(1) $$\dot{x}\left(t\right)=f\left(x\left(t\right)\right),$$

where $x\left(t\right)\in R^{n}$ denotes the system state. In SINDy, time‐series measurements are arranged into a data matrix X, and the corresponding time derivatives are collected in $\dot{X}$. A library of candidate nonlinear functions,

(2) $$\Theta \left(X\right)=\left[1,\;X,\;X^{P_{2}},\;\sin\left(X\right),\;\cos\left(X\right),\;\cdots \right],$$

is then constructed, and the dynamics are approximated as

(3) $$\dot{X}\approx \Theta \left(X\right)\Xi ,$$

where Ξ is a sparse coefficient matrix. In the original formulation, the coefficients are identified by seeking the sparsest model that still explains the measured derivatives, which may be written in idealized form as

(4) $$\min_{\Xi }\;∥\dot{X}{-\Theta \left(X\right)\Xi ∥}_{2}^{2}+\lambda {∥\Xi ∥}_{0},$$

or, in practice, by sequential thresholded least squares and related sparse regression procedures. This step distinguishes SINDy from ordinary nonlinear regression. The aim is not only to fit the data, but to select a small number of active terms from a much larger candidate set. The result is an interpretable model that is usually easier to analyse and validate than a black‐box predictor.

**FIGURE 1:**
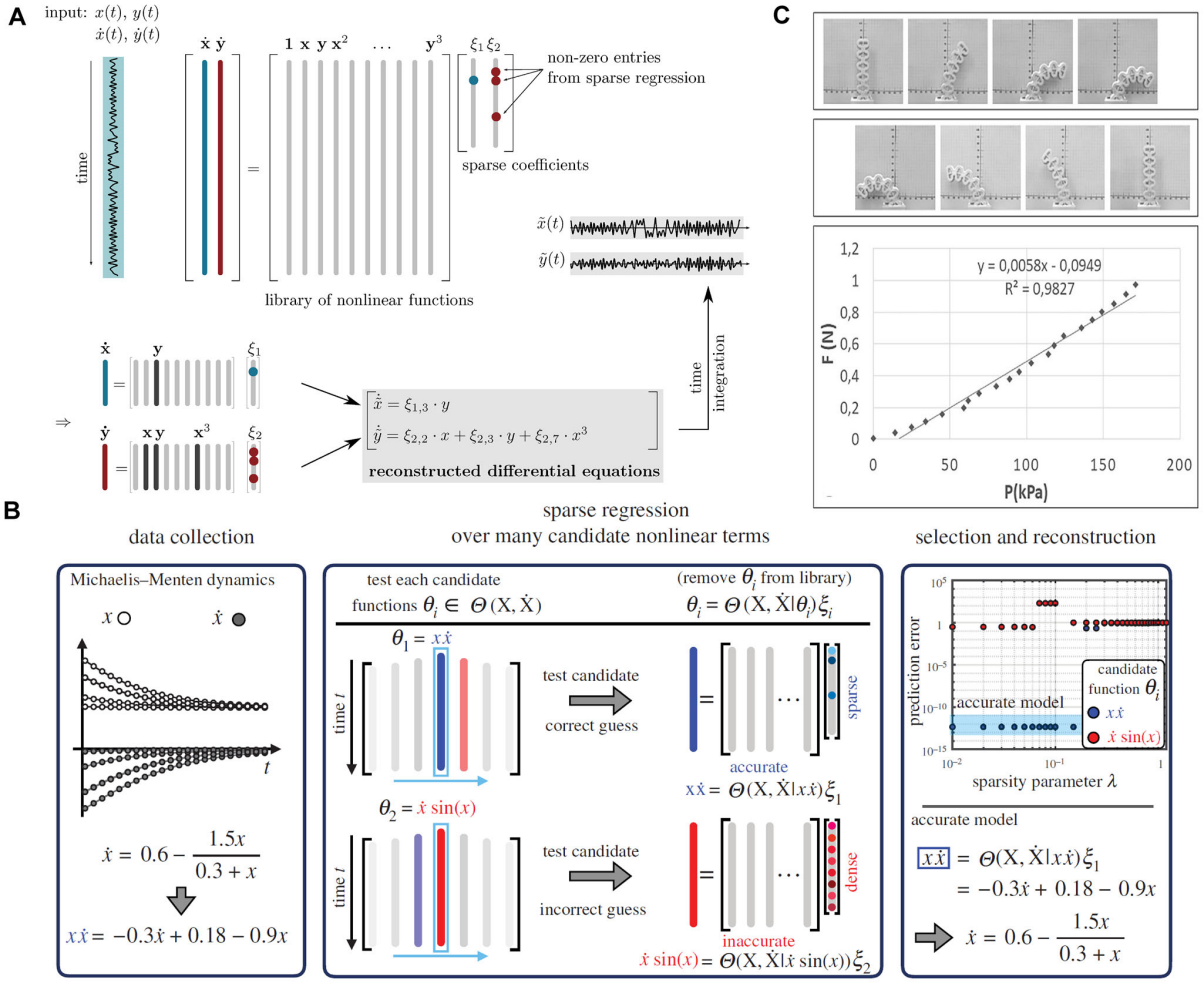
Sparse equation discovery and its relevance to soft robotics. (A) SINDy workflow for identifying sparse governing equations from measured state trajectories using a nonlinear candidate‐function library and sparse regression. Reproduced with permission under the terms of the Creative Commons CC BY license 4.0 [62]. Copyright 2019, MDPI. (B) Illustration of sparse regression and model selection, showing how candidate nonlinear terms are tested, removed, and reconstructed to obtain an accurate but parsimonious model. Reproduced with permission [63]. Copyright 2020, The Royal Society. (C) Soft‐robotic example from [61], showing representative configurations of a reconfigurable hybrid soft gripper together with the experimentally fitted relationship between actuation pressure and output force. Reproduced with permission under the terms of the Creative Commons CC BY license 4.0 [61]. Copyright 2026, Elsevier.

This property is especially attractive for soft manipulators. Their behavior arises from distributed deformation, pressure‐driven actuation, viscoelastic effects, geometric nonlinearity, and, in many tasks, intermittent contact with the environment. High‐fidelity continuum models can describe these effects in principle, but they are often too costly for online estimation or control [60]. SINDy provides an alternative reduced‐order description. Instead of resolving the full continuum field, it can identify reduced‐order dynamics directly in a physically meaningful state space, such as tip motion, modal coordinates, curvature variables, chamber pressures, or local deformation observables. For soft robotic systems, this creates a practical middle ground between first‐principles models that are difficult to calibrate and black‐box surrogates that offer limited physical insight [61].

Figure 1 illustrates this progression at three related levels. Figure 1A presents the canonical SINDy pipeline, in which measured trajectories are expanded into a candidate library and then filtered through sparse regression to recover a compact governing model. Figure 1B makes this idea more concrete by showing how candidate terms are retained or discarded, and how the final model is chosen by balancing accuracy against sparsity. Figure 1C then shows how this idea extends to soft robotics, where sparse equation discovery can support the design and grasp planning of a reconfigurable hybrid soft gripper by linking actuation, configuration, and output force in a compact model.

Another advantage of SINDy is that the discovered model remains explicit. Unlike generic neural predictors, the identified equations reveal which nonlinear couplings appear necessary to reproduce the measured response. The model should also help explain how actuation, deformation, loading, and dissipation interact. In this setting, SINDy is more than a regression tool. It provides a way to extract low‐order structure from data and express it in a form that can still support system identification, stability analysis, and control design [64].

Several extensions have broadened its use in robotics. Brunton et al. [65] extended the original formulation to controlled systems by incorporating exogenous inputs into the candidate library. In this setting,

(5) $$\dot{x}\left(t\right)=f\left(x\left(t\right),u\left(t\right)\right),$$

where u(t) denotes the external control input applied to the system at time t, such as chamber pressure in soft pneumatic systems. The regression problem then becomes

(6) $$\dot{X}\approx \Theta \left(X,U\right)\Xi ,$$

where U collects the measured input trajectories and Θ(X,U) may include both pure input terms and state–input coupling terms. This is important for soft manipulators because actuation is rarely autonomous. Pressure commands, tendon tensions, or magnetic inputs must appear explicitly in the model if the result is to be useful for feedback control or model predictive control (MPC). Ensemble‐SINDy improves robustness in noisy and data‐limited settings by aggregating models obtained from repeated resampling, providing term‐selection statistics and a practical measure of structural uncertainty [66]. This is particularly relevant in soft robotic experiments, where sensor drift, noisy derivative estimates, and limited repeatability are still common. SINDy‐PI, where PI denotes Parallel Implicit regression, extends sparse discovery to implicit dynamics. Rather than enforcing an explicit form $\dot{x}=f\left(x\right)$, it seeks a sparse relation of the form

(7) $$\Theta (x,\dot{x},u)\;\xi =0,$$

which is useful when the governing equations cannot be written cleanly in explicit state‐space form [63]. Such cases arise naturally in soft systems with pressure‐flow coupling, constrained internal variables, or nonlinear constitutive effects.

Recent reviews [67, 68, 69] in soft robotics have pointed to a growing need for explicit reduced‐order models that can support control. High‐fidelity continuum models are often too slow for online use, while unconstrained black‐box surrogates can be difficult to analyse and unreliable under extrapolation. In this context, the main appeal of SINDy is its modeling logic. It offers a direct way to turn measured soft‐robot behavior into compact governing equations rather than complicated predictors.

#### 2.1.1 Practical Viability and Limitations

For soft manipulators, the main limitation of SINDy is more fundamental than sensitivity to derivative estimates or library selection. Its sparse representation is credible only when the selected state contains the variables needed to make the dynamics approximately Markovian. This condition can be reasonable for low‐dimensional free‐motion models based on curvature, modal coordinates, chamber pressure, or end‐effector motion. It becomes much harder to justify when the response depends on unmeasured loading history, viscoelastic memory, internal pressure redistribution, or changing contact state. If these effects are omitted from the state, the same measured configuration and input may produce different state derivatives. In that case, no single‐valued sparse vector field of the form $\dot{x}=f\left(x,u\right)$ can represent the observed behavior consistently.

The sparsity assumption should therefore be treated as regime‐dependent in soft robotics. Within a fixed loading or unloading phase, or within a repeatable free‐motion regime, a small number of active terms may provide a useful reduced model. Across loading reversal, contact onset, sliding, detachment, and payload changes, the active terms may change and a single global sparse equation may lose physical meaning. Adding hysteretic internal states, delay coordinates, or mode indicators can restore a sparse description in some cases, although this also increases the state dimension and weakens the original simplicity of the method. Ensemble‐SINDy can improve term‐selection robustness under noisy and limited data, while SINDy‐PI can identify implicit and rational relations, but neither method resolves missing state information or unmodeled regime switching [63, 66, 67].

Our judgement is that SINDy is practically viable for interpretable, low‐order, and regime‐specific models when the system is sufficiently excited and the important memory variables are measured or reconstructed. It is less suitable as a single global model for contact‐rich soft manipulation, strong hysteresis, or distributed deformation with sparse sensing. In these cases, mode‐dependent SINDy, latent‐state augmentation, or a hybrid model with an analytical mechanical backbone is more defensible than increasing the polynomial library until the training error becomes small.

### 2.2 Operator‐Theoretic Modeling via Koopman learning

Koopman operator theory provides another way to model nonlinear systems from data [70]. Rather than identifying the governing equations term by term, it represents the nonlinear dynamics in a lifted observable space where the evolution is approximately linear. Figure 2A summarizes this idea: the nonlinear soft‐robot dynamics is lifted into a higher‐dimensional coordinate system, so that prediction and control can be handled with linear operators. Although the original dynamics remain nonlinear in the physical state, the lifted representation can often be approximated by a finite‐dimensional linear system that is more suitable for prediction, estimation, and control. For soft manipulators, this is attractive because it allows the use of linear control tools without relying on a single globally valid linearization around one operating point [71, 72].

**FIGURE 2:**
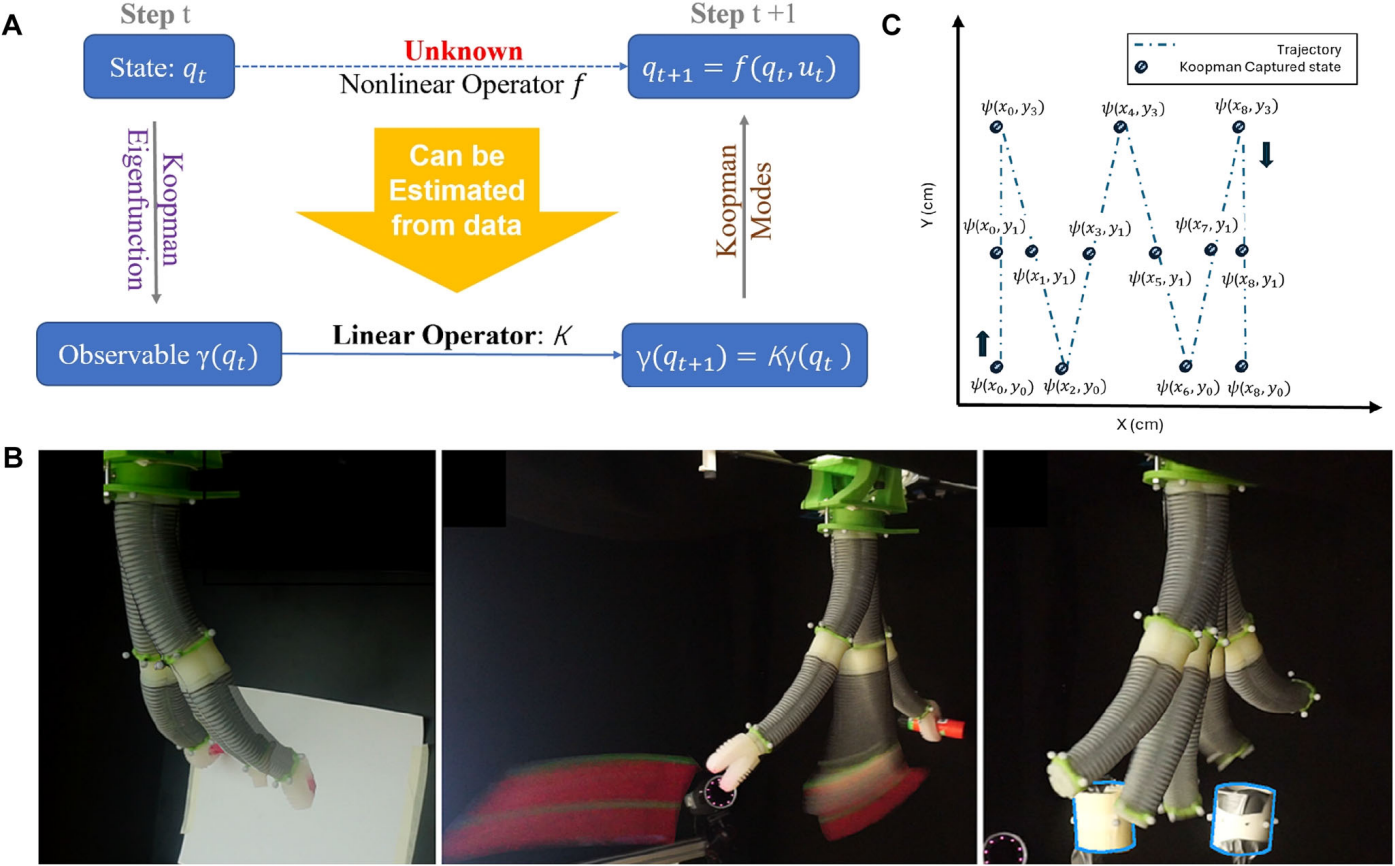
Koopman‐based modeling for soft robots. (A) Koopman lifting maps nonlinear state dynamics into a lifted observable space governed by a linear operator. Reproduced with permission under the terms of the Creative Commons CC BY license 4.0 [74]. Copyright 2023, Springer Nature. (B) Representative deformation configurations of a soft robotic arm under different actuation conditions. Reproduced with permission under the terms of the Creative Commons CC BY license 4.0 [75]. Copyright 2023, Wiley‐VCH. (C) Physical trajectory and corresponding Koopman‐captured states, illustrating the lifted representation of system evolution. Reproduced with permission under the terms of the Creative Commons CC BY license 4.0 [76]. Copyright 2026, Wiley‐VCH.

Consider a discrete‐time nonlinear system

(8) $$x_{k+1}=f\left(x_{k},u_{k}\right),$$

where $x_{k}\in R^{n}$ denotes the system state and $u_{k}\in R^{m}$ denotes the control input. Koopman‐based modeling introduces a lifting map ψ(·) that transforms the original state into a higher‐dimensional observable vector,

(9) $$z_{k}=\psi \left(x_{k}\right),$$

where ψ(·) denotes a vector of observables and $z_{k}$ represents the lifted state. Under this representation, the Koopman operator acts linearly on the observables, giving

(10) $$z_{k+1}=Kz_{k},$$

for autonomous dynamics. For controlled systems, one commonly seeks a finite‐dimensional approximation of the form

(11) $$z_{k+1}\approx Az_{k}+B_{z}u_{k},$$

together with a reconstruction map

(12) $$x_{k}\approx Cz_{k}.$$

Given lifted snapshot pairs, the matrices A and $B_{z}$ are usually identified by least squares or extended dynamic mode decomposition with control,

(13) $$\min_{A,B_{z}}\sum_{k}{\left\|z_{k+1}-Az_{k}-B_{z}u_{k}\right\|}_{2}^{2}.$$

In practice, the observable map may be chosen from hand‐crafted basis functions, delay coordinates, or learned dictionaries, and the finite‐dimensional linear predictor is then identified from lifted trajectory data. In practice, the method works only when the chosen observables lead to a useful finite‐dimensional approximation.

Figure 2A illustrates the basic idea of Koopman lifting for control. The nonlinear state evolution $q_{t+1}=f\left(q_{t},u_{t}\right)$ is mapped through observable functions into a lifted space, where the dynamics can be represented by a linear Koopman operator. Figure 2B shows representative deformations of a soft robotic arm under different actuation conditions, highlighting the strong nonlinear and configuration‐dependent behavior of soft systems. Figure 2C illustrates how states sampled along a physical trajectory can be represented by Koopman observables, providing a lifted description of the system evolution. These examples motivate Koopman‐based modeling for soft robotics, where nonlinear physical dynamics can be represented in a form that is more convenient for prediction and control. Such lifted models can be directly integrated with linear MPC, Linear–Quadratic Regulator (LQR), and observer design [71, 73].

This control‐oriented character is a key reason why Koopman methods have attracted attention in soft robotics. Bruder et al. [71] demonstrated that a pneumatic soft robot arm can be modeled in lifted coordinates and controlled using model predictive control, showing that Koopman identification can yield explicit linear models that outperform more opaque black‐box predictors in closed‐loop use. In later work, Bruder et al. [77] further developed Koopman‐based control formulations for soft robots, showing that these models are useful not only for prediction but also for controller design. Haggerty et al. [78] extended the Koopman perspective to a helically actuated inertial soft robotic arm, where the combination of Hankel‐based lifting, spectral analysis, and linear control enabled modeling, reduction, and control in a regime that is more dynamic than the quasi‐static settings often studied in soft robotics. More recent work has explored Koopman‐based shape control and offset‐free predictive control for soft manipulators [73, 79]. These studies suggest that the method is most useful when the goal is not only trajectory fitting, but also controller integration.

A recent study also reported quantitative results that support this point. Han et al. reported that, for 2D soft robots, three Koopman variants improved overall modeling accuracy by 32.98%, 33.42%, and 35.77%, while prediction accuracy improved by 69.54%, 71.67%, and 73.71%, respectively, relative to existing state‐space models. For 3D soft robots, the corresponding modeling improvements were 13.94%, 29.24%, and 37.48%, with prediction gains of 8.92%, 26.01%, and 36.76% [80]. These results suggest that Koopman lifting can offer practical gains in soft‐robot modeling. Its value is not only conceptual. With a suitable lifting design, it can improve prediction while keeping a model form that is convenient for control.

Koopman lifting changes the focus of modeling for soft manipulators. Instead of writing the dynamics directly in physical variables, one has to design a suitable observable space [81]. Once an observable space is chosen, the remaining dynamics become linear in form, and this greatly simplifies online optimization. Instead of repeatedly solving nonlinear prediction models at run time, the controller can operate on matrix multiplications and quadratic objectives. This can reduce computational burden and make timing more predictable, which is important for real‐time soft robotic control, especially when pressure regulation, interaction forces, and changing configurations must be handled at high update rates. In this sense, Koopman methods complement equation discovery. While SINDy seeks a sparse explicit vector field in the original variables, Koopman methods accept a higher‐dimensional representation in exchange for linear evolution and control compatibility.

#### 2.2.1 Practical Viability and Limitations

For soft manipulators, observable selection is difficult because the measured configuration usually represents only part of the system state. Chamber pressure, material relaxation, local strain, contact state, and distributed velocity may all affect future motion without being directly available to the lifting map. Delay coordinates or learned observables can partly compensate for this partial observability, but they also increase the lifted dimension and the amount of data required for identification.

The finite‐dimensional linear representation is also usually local to the operating conditions represented in the training data. Free motion, contact onset, sliding, load‐bearing deformation, and contact release may correspond to different transition maps. A single global Koopman model can represent these regimes only by using a sufficiently rich observable space, which may become too large for reliable identification and real‐time optimization. Soft‐robot studies have shown that Koopman models can support effective model predictive control and rapid dynamic manipulation when training data cover the intended motions [71, 82]. These results support their control value, while they do not establish that one lifting remains valid across arbitrary contact conditions, payloads, or robot specimens.

Our judgement is that Koopman learning is practically strong when the main objective is short‐horizon, control‐ready prediction over a bounded operating region. It is especially useful when repeated trajectories can be collected and when the controller benefits from a linear lifted model. It is less convincing for constitutive interpretation, strongly switching contact dynamics, and deployment far outside the training distribution. In such settings, local Koopman models, mode‐dependent lifting, online correction, or a physics–Koopman residual structure is more appropriate than a single global lifted system.

### 2.3 Continuous‐Time Latent Dynamics via Neural ODEs

Neural Ordinary Differential Equations (Neural ODEs) model system dynamics in continuous time directly from data. Instead of describing state evolution with a finite stack of discrete layers, Chen et al. [39] modeled the state derivative with a neural network and recovered the trajectory by integrating the learned vector field over time. As illustrated in Figure 3A, Neural ODEs represent system evolution through a continuous‐time latent dynamics model. The observed state is first mapped by an encoder into a latent representation, whose evolution is governed by a neural ordinary differential equation, and the resulting latent state is then decoded to reconstruct the system trajectory. This formulation provides a continuous‐time representation that is well suited to smoothly evolving dynamical systems and can naturally accommodate variable integration horizons and irregular sampling.

**FIGURE 3:**
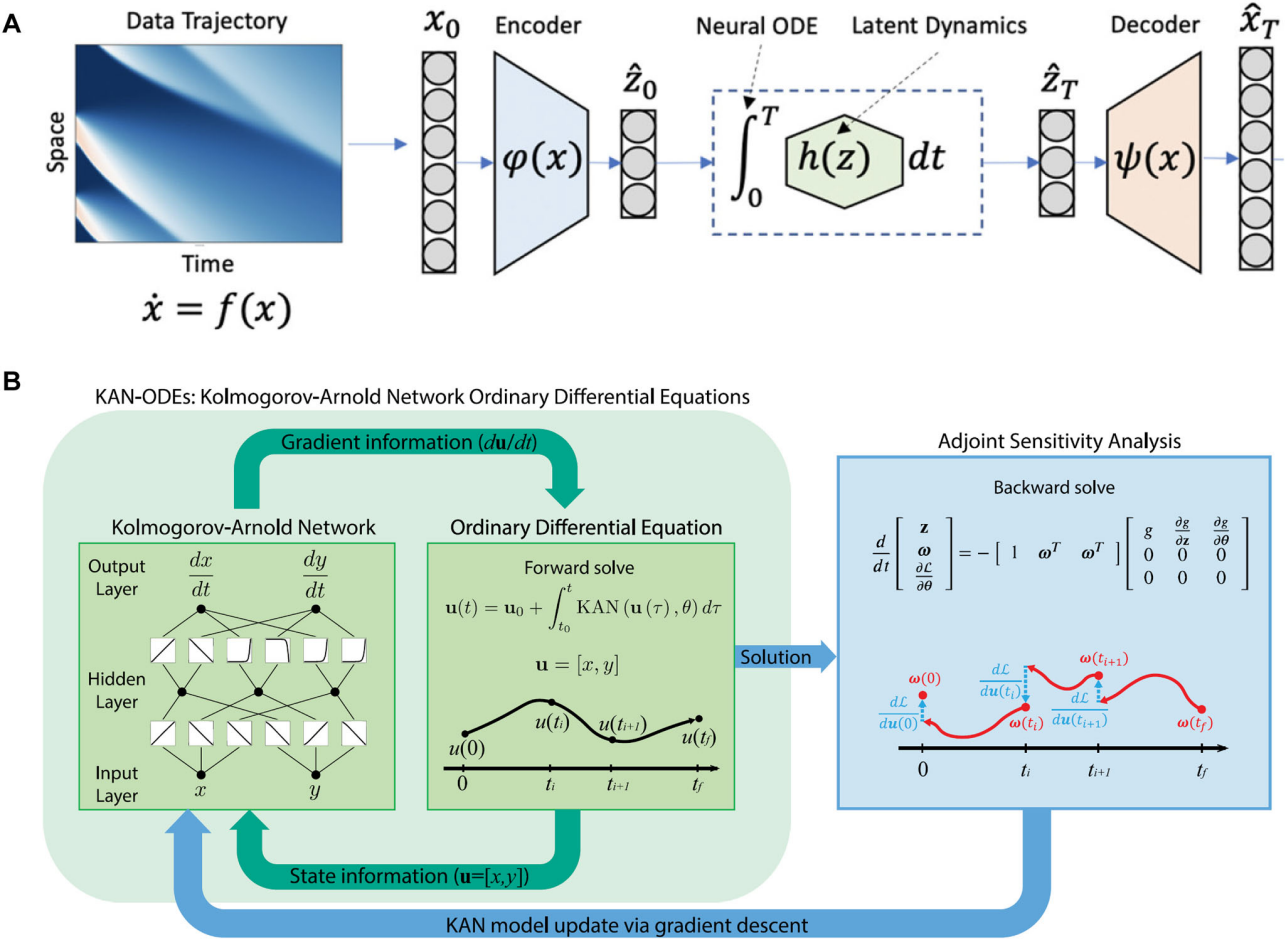
Continuous‐time neural dynamics for soft‐robot modeling. (A) Latent Neural ODE architecture with encoder, continuous latent dynamics, and decoder. Adapted with permission under the terms of the Creative Commons CC BY license 4.0 [85]. Copyright 2023, Springer Nature. (B) KAN‐ODE framework using a Kolmogorov–Arnold network with forward integration and adjoint sensitivity analysis. Reproduced with permission [86]. Copyright 2024, Elsevier.

Consider a continuous‐time nonlinear system

(14) $$\dot{x}\left(t\right)=f_{\theta }\left(x\left(t\right),u\left(t\right),t\right),$$

where $x\left(t\right)\in R^{n}$ denotes the system state, $u\left(t\right)\in R^{m}$ denotes the control input, and $f_{\theta }$ is a neural network with trainable parameters θ. Given an initial condition $x(t_{0})$, the state at a later time is obtained by numerical integration,

(15) $$x\left(t_{1}\right)=x\left(t_{0}\right)+\int_{t_{0}}^{t_{1}}f_{\theta }\left(x\left(t\right),u\left(t\right),t\right)\;dt.$$

In practice, the learned dynamics may be defined either in the physical state directly or in a latent space. When the state is only partially observed, an encoder can map measurements into latent variables z, the latent dynamics can then be evolved through the ODE solver, and a decoder reconstructs the measured outputs. This allows Neural ODEs to represent both explicit state‐space models and learned latent dynamical systems [83].

To reduce the high memory cost associated with standard backpropagation through continuous dynamics, the Neural ODE framework proposed by [39] is typically trained using the adjoint method [84]. This avoids storing all intermediate states along the forward trajectory, as would be required in a conventional deep network. Let L denote the loss function comparing the Neural ODE prediction with observed data, and let a(t)=∂L/∂z(t) denote the adjoint state, which measures how sensitively the loss depends on the hidden state z(t) at time t. The adjoint dynamics satisfy

(16) $$\frac{da(t)}{dt}=-a{\left(t\right)}^{⊤}\frac{\partial f_{\theta }\left(z\left(t\right),u\left(t\right),t\right)}{\partial z},$$

and the gradient with respect to the network parameters is given by

(17) $$\frac{dL}{d\theta }=-\int_{t_{1}}^{t_{0}}a{\left(t\right)}^{⊤}\frac{\partial f_{\theta }\left(z\left(t\right),u\left(t\right),t\right)}{\partial \theta }\;dt.$$

Rather than differentiating through a fixed sequence of layers, Neural ODEs compute parameter gradients by solving an adjoint differential equation backward in time.

For soft manipulators, the Neural ODE framework is useful because it represents time and hidden dynamics explicitly. Soft robotic motion is shaped by continuously varying pressure inputs, viscoelastic relaxation, distributed deformation, and delayed internal response. These features make the dynamics strongly time‐dependent and often difficult to describe with fixed discrete update rules. Neural ODEs provide a natural description of such behavior by learning the continuous‐time flow. Rather than predicting the next state through a single prescribed update, the model learns how the state changes at each instant, and the trajectory is recovered by integration over time. This is particularly useful for soft systems whose behavior evolves across multiple time scales.

This framework is also useful for soft manipulator modeling because it separates the learned dynamics from the discretization used in simulation. In a standard recurrent or feedforward predictor, the temporal step size is effectively built into the network architecture. In a Neural ODE, the vector field remains the same while the integration grid can change. This is useful when data are collected at different sampling rates, when sensing is asynchronous, or when the same model must support both offline simulation and online rollout. For soft robots, where experiments may involve different actuation rates, variable controller frequencies, or missing sensor data, this flexibility is useful in practice [39].

Figure 3B further shows the KAN‐ODE framework, in which a Kolmogorov–Arnold network parameterizes the continuous‐time vector field. The state dynamics are propagated through forward ODE integration, while adjoint sensitivity analysis provides gradients for updating the KAN parameters.

The framework is also useful because it connects black‐box sequence learning with more structured dynamical modeling. Roehrl et al. [87] showed that continuous‐time learned dynamics can be combined with prior mechanical structure by embedding Lagrangian knowledge into a physics‐informed neural modeling framework. Although this work was not developed specifically for soft manipulators, the same idea is relevant to soft robotic systems, where one often has partial mechanical knowledge but not a complete constitutive model. More recent work has explored alternative parameterizations of the continuous‐time vector field. Koenig et al. [86] proposed KAN‐ODEs, which replace standard multilayer perceptrons with Kolmogorov–Arnold networks in the Neural ODE backbone, showing that continuous‐time learning can also be combined with more structured and potentially more interpretable function classes. These developments suggest that Neural ODEs can be used not only as generic predictors, but also as a modeling layer that incorporates additional physical structure.

For soft manipulators, this flexibility matters because the state representation is often uncertain. In some cases, the most useful variables may be tip position, curvature, chamber pressure, or a small number of modal coordinates. In other cases, the relevant dynamics may live in a latent space that cannot be written down directly from first principles. Neural ODEs can accommodate both settings. When low‐dimensional physical coordinates are available, the model can be trained directly in those variables. When the measurements are high‐dimensional or incomplete, the ODE can instead govern an encoded latent state. This makes the framework particularly attractive for soft robotic systems in which distributed deformation is difficult to observe fully, yet one still seeks a compact dynamical model for forecasting or control.

#### 2.3.1 Practical Viability and Limitations

The continuous‐time form of a Neural ODE is well matched to smoothly evolving deformation, but several important soft‐robot phenomena are only piecewise smooth. Contact onset, stick–slip transition, valve switching, pressure saturation, and loading reversal can produce sharp changes that a single smooth neural vector field may smear across time. The model may then obtain a low average trajectory error while predicting incorrect contact timing or peak force, which can be more important for control than the average state error.

Soft manipulators can also contain several time scales. Pneumatic transients may evolve faster than viscoelastic relaxation, while the mechanical response may become stiff near contact or under high loading. In these cases, the ODE solver may require many internal steps, making the model slower and less predictable than its compact network representation suggests. A model trained on short trajectories may also produce unstable or non‐dissipative long‐horizon rollouts. Stabilised and dissipative neural dynamical models have been introduced specifically to address these problems [67, 88, 89].

Our judgement is that Neural ODEs are practically viable for smooth free‐motion dynamics, irregularly sampled trajectories, and latent‐state modeling when time continuity is important. Their value is weaker for high‐rate control involving repeated contact transitions, stiff pressure–structure dynamics, or long prediction horizons unless stability, dissipation, switching modes, or mechanical structure are included explicitly. In these cases, a structured or hybrid Neural ODE is more credible than an unconstrained neural vector field.

### 2.4 Physics‐Informed Neural Networks

Physics‐Informed Neural Networks (PINNs) combine data‐driven learning with known physical laws during training. Rather than relying only on input‐output supervision, PINNs include governing equations, conservation laws, or constitutive constraints in the loss function, so that the model is trained to match both measured data and physical constraints [47]. Figure 4 illustrates several ways in which physical knowledge can be incorporated into neural modeling. In Figure 4A, a PINN reconstructs the solution of a nonlinear partial differential equation (PDE) from sparse observations while remaining consistent with the governing equation. Automatic differentiation provides the temporal and spatial derivatives required to evaluate the physical residual during training.

**FIGURE 4:**
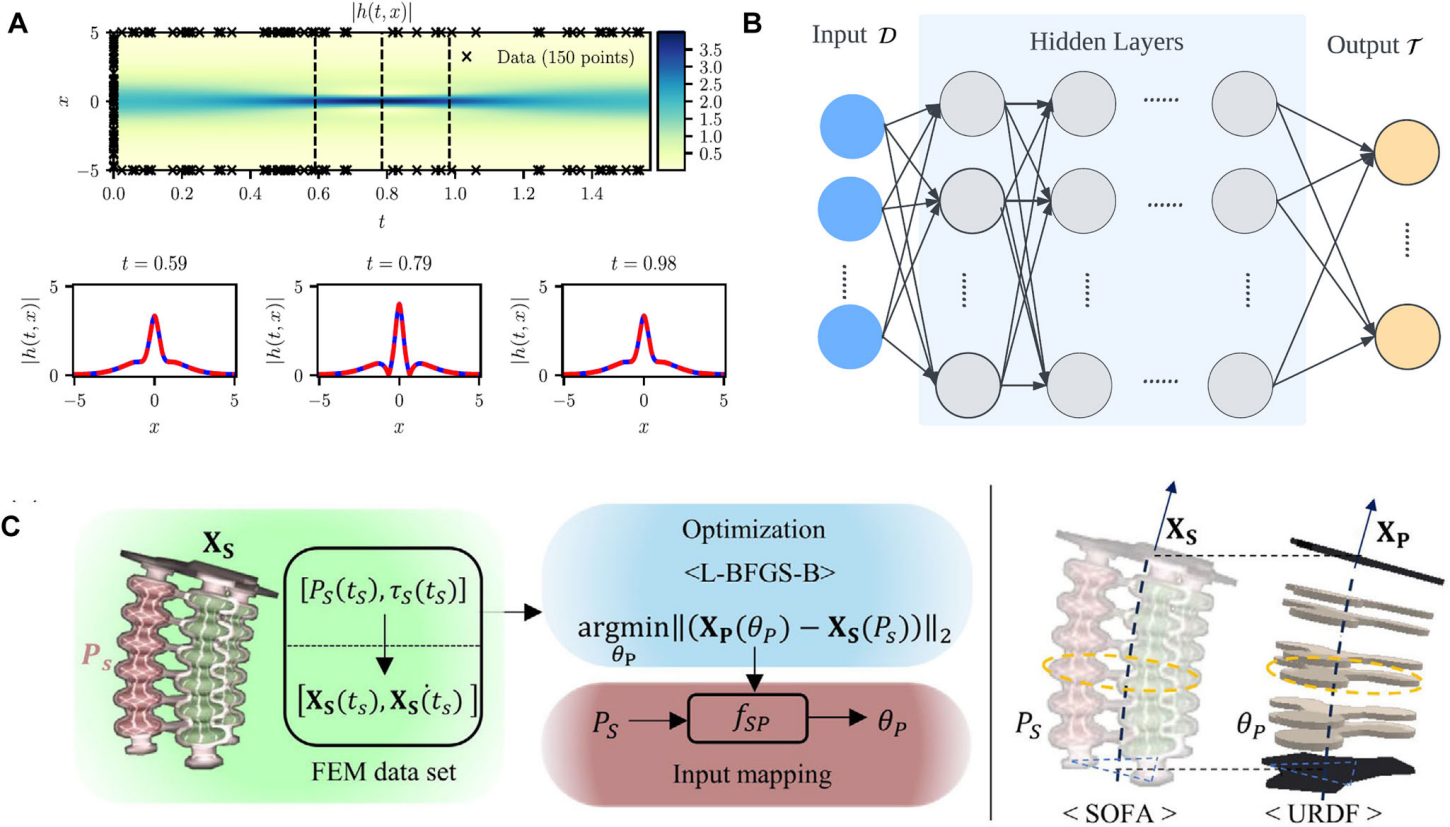
Physics‐informed neural modeling for soft robotic systems. (A) PINN‐based solution of a nonlinear partial differential equation from sparse observations, including field reconstruction and comparison between exact and predicted profiles. Reproduced with permission [47]. Copyright 2019, Elsevier. (B) Neural‐network mapping from input data to target system quantities. Reproduced with permission under the terms of the Creative Commons CC BY license 4.0 [90]. Copyright 2024, Wiley‐VCH. (C) Physics‐informed parameter identification between FEM‐based Simulation Open Framework Architecture (SOFA) and Unified Robot Description Format (URDF) models through configuration matching. Adapted with permission under the terms of the Creative Commons CC BY license 4.0 [91]. Copyright 2026, Wiley‐VCH.

In a standard setting, a neural network is used to approximate the state or field variables of interest,

(18) $$\hat{x}\left(t\right)=N_{\theta }\left(t,u\right),$$

or, more generally, a spatiotemporal field,

(19) $$\hat{y}\left(s,t\right)=N_{\theta }\left(s,t,u\right),$$

where θ denotes the trainable parameters, u represents system inputs, and s denotes the spatial coordinate when distributed deformation is considered. Following the standard PINN formulation, the network output is substituted into the governing differential equation to define a physics residual. For a general nonlinear equation written abstractly as

(20) F[y](s,t;u)=0,

the residual induced by the neural approximation is

(21) $$r_{\theta }\left(s,t\right)=F\left[\hat{y}\right]\left(s,t;u\right).$$

The network is trained not only by minimizing mismatch with observations, but also by penalizing the residual of the governing equations,

(22) $$L=L_{\mathrm{data}}+\lambda_{\mathrm{phys}}L_{\mathrm{phys}}+\lambda_{\mathrm{bc}}L_{\mathrm{bc}},$$

where $L_{\mathrm{data}}$ measures agreement with experimental or simulated data, $L_{\mathrm{phys}}$ enforces the differential equations or mechanical constraints, and $L_{\mathrm{bc}}$ accounts for boundary or initial conditions. Equation (22) captures the same balance shown in Figure 4. In Figure 4A, for instance, the learned field is constrained to remain consistent with the governing PDE while matching sparse observations. Automatic differentiation allows the temporal and spatial derivatives in $r_{\theta }\left(s,t\right)$ to be computed directly from the network, so the governing equations can be imposed during training as differentiable constraints rather than through a separate numerical solver.

For soft manipulators, this formulation is useful because the modeling problem is rarely purely data‐driven or purely first‐principles. Continuum mechanics, rod theory, beam models, or finite‐element formulations often provide a meaningful description of deformation, stress, and actuation response, but these models may be too costly for repeated calibration, real‐time prediction, or online control. At the same time, purely black‐box predictors can fit data well while violating force balance, energy dissipation, geometric compatibility, or boundary conditions. PINNs allow the model to adapt to data while still respecting the mechanical structure that is already known. This is particularly valuable in soft robotics, where sensing is often sparse, internal states are only partially measurable, and experiments are expensive to repeat.

The deformation of a soft body is spatially distributed, strongly nonlinear, and often affected by viscoelasticity, actuation delay, and environmental contact. In many cases, one does not need a full high‐fidelity field solution at every point in space and time, but one still wants the learned model to remain compatible with mechanics. A PINN can do this by learning a reduced or surrogate mapping while constraining it to satisfy the underlying physical relations. Here the method helps in two ways: it predicts system behavior and regularizes learning when observations alone are not enough to identify a reliable model.

Recent studies have applied this idea to soft robotic systems. Sun et al. [92] used a physics‐informed recurrent neural framework to model soft pneumatic actuators, showing that mechanical priors can improve prediction under nonlinear and hysteretic conditions. Separately, Figure 4B, reproduced from Liu et al. [90], illustrates a neural‐network mapping from input data to target system quantities, with physical knowledge incorporated through the model formulation and training objective. Figure 4C shows a physics‐informed parameter‐identification framework in which FEM‐generated states are matched to a reduced URDF representation through configuration‐based optimization. In related work, Khoshbakht et al. [93] applied physics‐informed learning to tendon‐driven soft robots for shape reconstruction and tip force estimation, which is particularly relevant to soft manipulators operating under partial sensing. These studies also indicate that physics‐informed learning can be used to estimate hidden variables that are difficult to measure directly, not just to reproduce trajectories.

PINNs can also be used in several ways. In some cases, the network approximates the full state or deformation field while satisfying partial differential equations or rod equations. In other cases, the network represents only the unknown part of the dynamics, while the known structure is imposed analytically. This matters for soft manipulator modeling because the available prior knowledge can vary widely across platforms. For one system, chamber pressure and curvature relations may be reasonably well understood. For another, only approximate energy or boundary constraints may be available. Here PINNs are useful because they can incorporate partial physics without requiring a fully specified constitutive model.

In soft robotics, PINNs are useful for more than data efficiency alone. More broadly, they fit the current interest in real‐time surrogates that preserve at least part of the underlying mechanics, especially when full nonlinear FEM is too costly and purely data‐driven mappings are too unconstrained [67]. They do not remove the modeling difficulty; instead, they move it into the design of a constrained function class.

#### 2.4.1 Practical Viability and Limitations

For soft manipulators, the main difficulty of PINNs is that the available physics may be both incomplete and numerically demanding. Loss balancing is not universally prohibitive. It can be manageable for smooth quasi‐static deformation, parameter identification, or field reconstruction when the governing equations and boundary conditions are reasonably accurate. The difficulty becomes much more serious when a single model must combine force balance, constitutive behavior, pressure dynamics, initial and boundary conditions, contact constraints, and sparse experimental measurements.

These loss terms can have very different magnitudes and gradient scales. Training may reduce the easiest residual while leaving task‐relevant errors, such as contact force or tip displacement, poorly resolved. Previous studies have shown that differential operators in the physics‐informed loss can produce poorly conditioned optimization problems and severe gradient imbalance [94, 95]. This issue is particularly relevant to soft robots because their equations often contain large deformation, stiff material response, several physical units, and strongly different temporal and spatial scales.

Incomplete physics creates a second problem. If the imposed constitutive law neglects hysteresis, friction, fabrication asymmetry, fluid–structure interaction, or contact switching, increasing the physics‐loss weight can push the network toward a physically tidy but experimentally incorrect solution. Reducing the physics weight avoids this bias, but it also weakens the claimed physical constraint. Standard PINNs are also poorly matched to discontinuous contact forces and changing boundary conditions unless domain decomposition, separate contact modes, inequality constraints, or hybrid residual terms are introduced.

Our judgement is that PINNs are practically useful for offline inverse problems, sparse field reconstruction, and surrogate modeling when the governing physics is smooth and credible. They are currently less convincing as a general replacement for numerical mechanics in contact‐rich, dynamic soft manipulation. For these problems, the cost and uncertainty of loss design can approach or exceed the difficulty of calibrating a reduced mechanical model. A more practical role is often to infer hidden fields, estimate a limited number of unknown parameters, or construct a fast surrogate around a validated physical model.

### 2.5 Field‐Level Surrogates via Neural Operators and Graph Simulators

Neural operators and graph‐based learned simulators provide another route for modeling soft manipulators when the quantity of interest is a distributed field rather than a small set of lumped coordinates. Unlike standard neural networks that learn mappings between finite‐dimensional vectors, neural operators learn mappings between function spaces. For a soft manipulator, this can be interpreted as a learned operator from actuation, material, geometry, or boundary‐condition fields to deformation, strain, stress, pressure, or contact‐force fields,

(23) $$G_{\theta }:a\left(s\right)\mapsto y\left(s\right),$$

where a(s) denotes an input field and y(s) denotes the predicted physical response field.

Deep Operator Networks (DeepONets) learn such operators through a branch network that encodes the input function and a trunk network that encodes the output location [40, 96]. Fourier Neural Operators (FNOs) instead parameterize the integral kernel in Fourier space, enabling efficient learning of families of parametric partial differential equations [41, 97, 98]. These methods are relevant to soft manipulators because many soft‐robot modeling problems are naturally field‐to‐field problems, such as pressure‐to‐shape prediction, geometry‐to‐deformation prediction, and material‐parameter‐to‐stress prediction. Compared with repeatedly solving a high‐fidelity finite‐element model, a trained operator surrogate can provide much faster predictions over a family of related configurations.

Graph neural network simulators provide a related but more mesh‐aware formulation. MeshGraphNets represent the simulated body as a graph whose nodes and edges carry local geometric and physical information, and then use message passing to propagate deformation and interaction dynamics [99, 100]. This is attractive for soft robotics because soft manipulators are often discretized by meshes, rods, particles, or finite elements, and their behavior depends strongly on local interactions, contact, and boundary conditions. Graph‐based simulators therefore offer a possible bridge between high‐dimensional numerical simulation and learned reduced‐order prediction.

#### 2.5.1 Practical Viability and Limitations

Neural operators and graph simulators are promising when broad simulation or experimental data are available over meaningful variations in geometry, actuation, loading, and material properties. Their main advantage is that they can amortize expensive field‐level simulation into a fast surrogate after training. However, they are not a simple substitute for mechanics. They usually require large and diverse field‐level datasets, and their generalization can degrade when the robot geometry, contact regime, material law, or sensing distribution changes beyond the training range. For soft manipulators, contact‐rich manipulation, hysteresis, frictional switching, and sparse sensing remain difficult because these effects are often poorly represented by a single smooth operator. In such cases, neural operators and graph neural network (GNN) simulators are most credible as part of a hybrid architecture, where they accelerate a validated physical model or provide a learned correction within a bounded operating domain.

### 2.6 Hybrid Physics–ML Models

Hybrid physics–machine learning models combine analytical structure with data‐driven flexibility within a single framework. Rather than viewing physics‐based and learning‐based methods as competing alternatives, hybrid models use them to describe different parts of the problem. The analytical component captures the mechanical structure that is already known, such as geometry, constraints, or low‐order dynamics, while the learned component accounts for effects that are difficult to model explicitly, including hysteresis, unmodeled compliance, actuator nonlinearities, parameter drift, and contact‐related errors.

A general hybrid formulation can be written as

(24) $$\dot{x}\left(t\right)=f_{\mathrm{phys}}\left(x\left(t\right),u\left(t\right)\right)+f_{\mathrm{learn}}\left(x\left(t\right),u\left(t\right);\theta \right),$$

where x(t) denotes the system state, u(t) denotes the control input, $f_{\mathrm{phys}}$ represents the known or assumed continuous‐time physical dynamics, and $f_{\mathrm{learn}}$ is a learned correction term parameterized by trainable parameters θ. In this additive form, the learned component accounts for the part of the state dynamics that is not adequately represented by the nominal physical model.

The learned component does not always appear as an additive correction to the state dynamics. Learning is instead used to estimate uncertain or effective physical quantities that are subsequently used within the physics‐based model. A representative parameter‐learning form can be written as

(25) $$y\left(t\right)=g_{\mathrm{phys}}\left(x\left(t\right),u\left(t\right),\pi_{\mathrm{eff}}\left(t\right)\right),\;\pi_{\mathrm{eff}}\left(t\right)=h_{\theta }\left(x(t),u(t)\right),$$

where y(t) denotes the model output, $g_{\mathrm{phys}}$ denotes a physics‐based input–state–output mapping, and $\pi_{\mathrm{eff}}\left(t\right)$ denotes a set of effective physical parameters estimated by the learned mapping $h_{\theta }$. Depending on the application, $\pi_{\mathrm{eff}}$ represent quantities such as effective stiffness, damping, or actuation coefficients. Mechanically meaningful structure is retained where it is reliable, while data‐driven components are used to represent the remaining uncertainty or model discrepancy.

For soft manipulators, this approach is particularly useful because neither pure mechanics nor pure learning performs well across all regimes. High‐fidelity continuum or finite‐element models can describe large deformation, distributed loading, and actuator response with strong physical grounding, but they are often computationally expensive, difficult to calibrate, and sensitive to uncertain material parameters. Purely learned models can be fast and expressive, yet they may require large datasets, extrapolate poorly, and provide limited physical transparency. Hybrid models offer a more workable way to handle this trade‐off. They keep the parts of the dynamics that are already understood and use the learned component for effects that remain difficult to derive from first principles [101].

This balance matters in soft robotics because the gap between theory and experiment is often substantial and varies across operating conditions [102, 103]. Material behavior may depend on fabrication variability, internal damping may change with operating history, chamber pressure may not map cleanly to deformation, and environmental contact can introduce regime‐dependent dynamics that are hard to encode analytically. A first‐principles model may capture the right structure but still miss the quantitative behavior, whereas a purely data‐driven model may fit the training trajectories without preserving the underlying mechanics of the system. Hybrid modeling offers a more practical middle ground. The physics‐based component keeps the model tied to physically meaningful behavior, while the learned component adjusts the prediction to match experimental observations [90].

Figure 5 shows two representative ways in which learning can reduce the gap between nominal physics and observed behavior. Figure 5A illustrates a Sim2Real framework in which a conditional learned mapping transforms simulated soft‐gripper states toward experimental observations under different contact conditions. Figure 5B shows a residual‐learning approach, where a learned residual force augments a physics‐based simulator to compensate for systematic model error.

**FIGURE 5:**
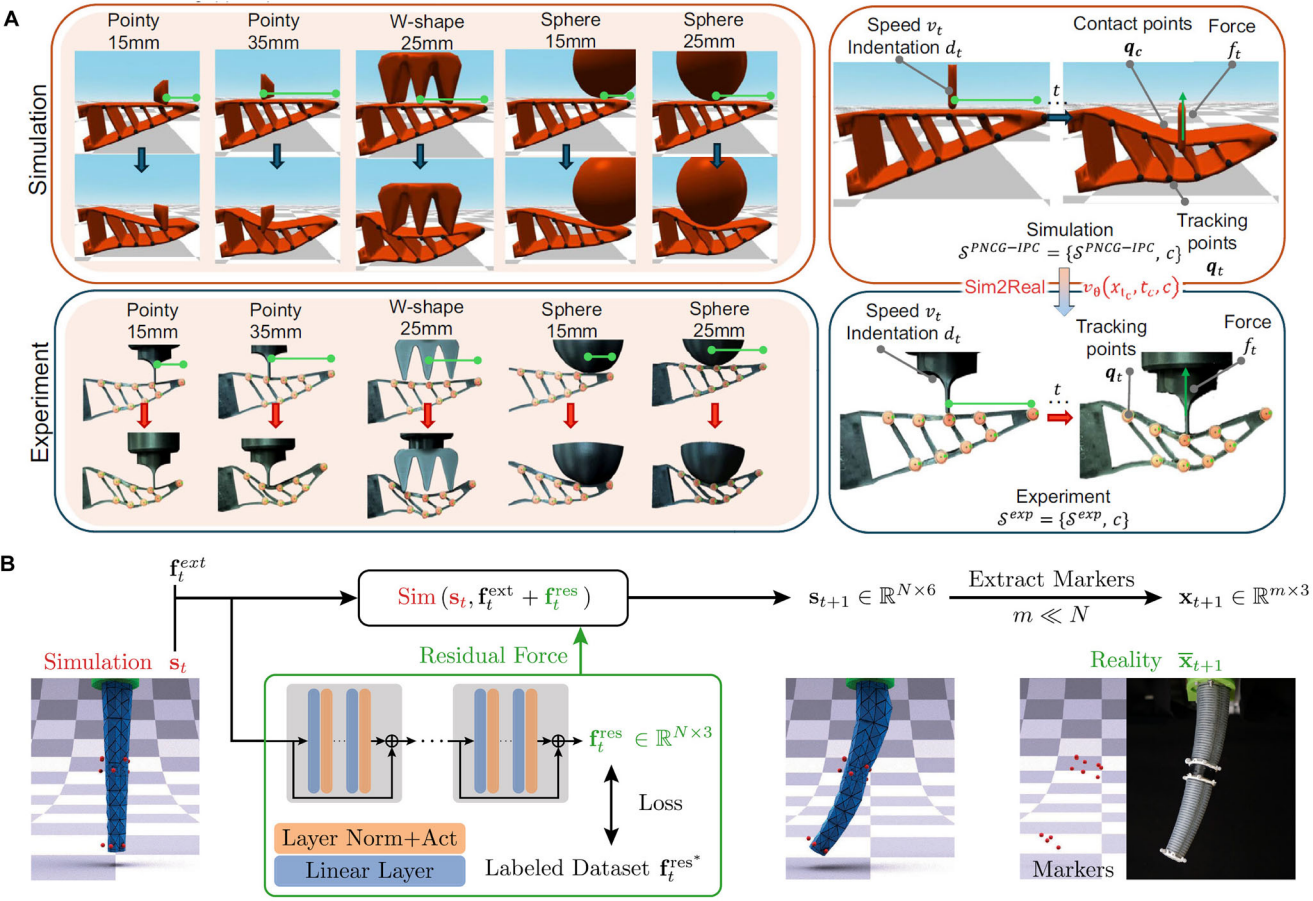
Hybrid physics–machine learning modeling for soft robotic systems. (A) Sim2Real framework for a fin‐ray soft gripper, where a learned conditional mapping transforms simulated soft‐body states toward real experimental observations under different contact conditions. Reproduced with permission under the terms of the Creative Commons CC BY license 4.0 [104]. Copyright 2026, Wiley‐VCH. (B) Residual‐learning framework in which learned residual forces augment a physics‐based simulator to reduce discrepancies between simulated and real soft‐body deformation. Reproduced with permission under the terms of the Creative Commons CC BY license 4.0 [102]. Copyright 2024, IEEE.

Recent studies have shown that this strategy can be useful in both modeling and control of soft robots. Tang et al. developed a learning‐based control framework for soft robot–environment interaction and reported that, in a horizontal wiping task, the root‐mean‐square errors in position and force tracking were 4.58 mm and 0.021 N, respectively, less than half the corresponding probabilistic inference for learning control (PILCO) benchmark values of 13.35 mm and 0.074 N [105]. This suggests that a structured surrogate model, when embedded in a predictive control architecture, can improve tracking in variable interaction settings rather than simply fitting trajectories offline. In a different direction, Zhang et al. introduced a gas–ribbon hybrid soft finger that operated below 35 kPa, achieved a maximum bending angle of approximately 210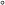, supported loads up to 1.25 kg, and increased stiffness by roughly three to six times across the bending range [106]. Fu et al. further combined tendon and pneumatic actuation in a hybrid‐driven soft manipulator, reporting an elongation of 198% and a maximum bending angle of 240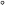 while enabling simultaneous stiffness and position modulation [107]. Ding et al. used a multiphysics finite‐element framework to simulate cable‐, Shape Memory Alloys (SMA)‐, and pneumatic‐actuated soft modules, reporting planar bending tip errors of 2.90% with solid cable elements and 5.97% with beam cable elements, while dual‐cable actuation reduced the error to about 1.65%; for an SMA‐driven module, the height‐change error was 4.4% [108]. Together, these studies suggest that hybridization can improve control accuracy, expand actuation capability, and reduce modeling error when a single modeling paradigm is not enough.

Hybrid models are also flexible in where the coupling is introduced. In some cases, the hybridization occurs at the level of state evolution, where the neural network learns a residual force, residual acceleration, or residual state derivative. In other cases, the hybridization occurs at the level of parameters, where the learned module estimates effective stiffness, damping, or actuation coefficients for use in a reduced‐order mechanical model. It may also occur at the level of latent representation, where physics constrains part of the state while learning governs hidden or unresolved components. This matters in soft manipulators because the main source of modeling error varies from platform to platform. For one platform, the main uncertainty may lie in actuation. For another, it may lie in contact, constitutive response, or sensing incompleteness. A good hybrid framework should place the learned component where the real uncertainty lies.

#### 2.6.1 Determining the Physics–Learning Boundary

The boundary between the analytical and learned components should be determined from the intended task and the observed model discrepancy, rather than from a fixed preference for either physics or learning [109]. The analytical component should retain relations that are well established, identifiable from the available measurements, relevant to control or safety, and computationally affordable at the required update rate [110]. For soft manipulators, these relations commonly include geometric compatibility, force balance, actuation limits, and the main elastic or dissipative structure. The nominal physical model should first be calibrated and tested across the intended operating range before a learned component is introduced [30, 33].

A practical way to locate the remaining uncertainty is to examine the one‐step prediction residual of the calibrated physical model. For a discrete‐time representation, this residual can be written as

(26) $$r_{k}=x_{k+1}^{\mathrm{meas}}-\Phi_{\mathrm{phys}}\left(x_{k},u_{k};\hat{\pi }\right),$$

where $x_{k+1}^{\mathrm{meas}}$ denotes the measured state at the next time step, $\Phi_{\mathrm{phys}}$ denotes the discrete‐time state transition induced by the calibrated physical model, and $\hat{\pi }$ contains the identified physical parameters [102, 109]. Here, $\Phi_{\mathrm{phys}}$ is distinguished from the continuous‐time vector field $f_{\mathrm{phys}}$ in Equation (24); it represents the corresponding one‐step prediction used for sampled‐data analysis and residual evaluation. If $r_{k}$ is comparable to measurement and identification noise and shows no repeatable dependence on the state, input, loading history, or contact condition, an additional learned component is unlikely to be justified. If the discrepancy can be explained by a small number of slowly varying quantities, such as stiffness, damping, or pressure‐to‐force gain, only these parameters should be updated. A learned residual is more appropriate when a repeatable nonlinear discrepancy remains after parameter calibration. Examples include hysteresis, viscoelastic rate effects, pneumatic delay, fabrication asymmetry, and contact‐dependent forces.

The location of the learned term should also follow the source of the discrepancy. Parameter learning is suitable for low‐dimensional drift [111], whereas force‐ or state‐residual learning is suitable for systematic model error. Mode‐dependent learning is needed when free motion, contact, sliding, and load‐bearing behavior follow different dynamics. Latent‐state learning should be reserved for cases where relevant internal variables cannot be measured or represented using the selected physical coordinates [39]. This division is reflected in residual‐physics models that learn corrective forces on top of a soft‐body simulator [102], and in hybrid soft‐robot models that retain a quasi‐static mechanical description while using neural components to represent nonlinear dynamics and pneumatic hysteresis [112].

The final boundary can be selected by comparing a nested set of candidate models, such as a physics‐only model, a parameter‐adaptive model, a residual hybrid model, and a fully learned model. One practical selection rule is

(27) $$\begin{array}{ccc} & & A^{★}=\underset{A\in H}{\arg\min}\;C_{\mathrm{learn}}\left(A\right)\;\mathrm{subject}\;\mathrm{to}\;E_{\mathrm{task}}\leq \varepsilon_{T},\\ & & V_{\mathrm{phys}}\leq \varepsilon_{P},\;t_{\mathrm{eval}}\leq t_{\max}, \end{array}$$

where $C_{\mathrm{learn}}$ measures the complexity of the learned component, $E_{\mathrm{task}}$ is the error in the task‐relevant outputs, $V_{\mathrm{phys}}$ measures violations of physical or safety constraints, and $t_{\mathrm{eval}}$ is the model evaluation time. Here, H denotes the set of candidate model architectures, while $\varepsilon_{T}$, $\varepsilon_{P}$, and $t_{\max}$ denote the allowable task error, physical‐constraint violation, and model‐evaluation time, respectively. The preferred boundary is therefore the least flexible learned component that satisfies the required task accuracy, physical consistency, and real‐time constraints on held‐out operating conditions. If the learned correction becomes comparable to, or larger than, the nominal physical contribution over most of the operating range, the physical model or state representation should be reconsidered instead of allowing the learned component to compensate for the entire system.

More broadly, hybrid methods support adaptation without discarding prior physical knowledge. Soft manipulator dynamics may shift over time due to material aging, pressure leakage, changing payloads, or accumulated wear. An analytical model can drift out of date unless it is recalibrated repeatedly, whereas a purely learned model may require a large amount of new data before it becomes reliable again. A hybrid model can update only the residual layer or effective parameters while keeping the physically meaningful base dynamics unchanged. This is particularly useful for systems that require continual refinement, online updating, or digital‐twin‐style synchronization between model and hardware [113].

Hybrid modeling also brings its own challenges. The interaction between the physical and learned components must be designed carefully. If the analytical backbone is too inaccurate, the learned module may end up compensating for nearly the entire system, in which case the benefit of including physical structure becomes limited. If the learned correction is too flexible, it can undermine the physical interpretation of the base model and make closed‐loop analysis harder. How the model splits known and unknown dynamics is therefore a central design choice. In soft robotics, where many effects are coupled and regime‐dependent, this allocation is rarely obvious. The success of a hybrid model depends not only on the quality of each component, but also on whether this decomposition matches the actual structure of the system [16, 114].

For this reason, hybrid models require deliberate architectural decisions rather than a simple combination of two modeling tools. The residual‐based procedure described above makes this division more explicit. Physics should retain the relations that remain reliable, task‐relevant, and necessary for control or safety, while learning should be limited to uncertain parameters, repeatable model residuals, or unresolved regime‐dependent effects. In this form, hybrid physics–machine learning can extend an incomplete mechanical model without allowing the learned component to obscure the physical structure that the model is intended to preserve.

#### 2.6.2 Differentiable Simulation and Modeling

Differentiable simulation refers to modeling frameworks in which the physics‐based dynamics of a robotic system are formulated such that gradients of states, controls, and model parameters can be computed through the simulation process [115]. In contrast to conventional numerical solvers that provide only forward predictions of motion, differentiable models expose the sensitivities of those predictions with respect to the underlying variables. This capability enables the seamless integration of physical models with optimization and learning algorithms, providing a natural bridge between data‐driven techniques and physics‐informed modeling. For soft manipulators, whose continuum, compliant, and nonlinear behaviors challenge both analytic modeling and pure machine learning, differentiable physics provides a principled way to embed physical structure while retaining flexibility to adapt to data [116].

The advantages of differentiable simulation stem from its ability to unify physical priors and data in an end‐to‐end computational framework. Because gradients can be propagated through the equations of motion, one can incorporate learning components that refine unknown or uncertain aspects of the model while preserving physically meaningful structure. This allows simulation‐based algorithms to solve control, estimation, and design problems directly through gradient‐based optimization, improving sample efficiency and enabling rapid adaptation to real‐world observations [117]. Moreover, the capacity to optimize over physical parameters, control inputs, and even morphological features in a consistent manner supports more integrated workflows than those traditionally used in robotics. For soft systems, where material parameters, damping characteristics, and actuation models often vary between fabricated specimens, differentiable modeling offers an efficient mechanism for identifying or updating these characteristics automatically using trajectory data [116].

Differentiable simulation has already enabled a range of notable applications in robotics. In control and trajectory optimization, differentiable model predictive control (MPC) and end‐to‐end policy learning have been demonstrated through frameworks that compute gradients directly through the dynamics, allowing task objectives to be optimised with respect to control inputs. Early examples include differentiable MPC for rigid‐body systems [117] and end‐to‐end differentiable physics for learning dynamical models and controllers [118]. For soft robots in particular, real‐time differentiable simulators such as ChainQueen [119] have shown that gradient‐based control can be performed directly on continuum, deformable dynamics. Beyond control, differentiable physics has proven powerful for co‐design and morphology optimization, where both physical parameters and geometric features can be optimized jointly with control policies. Differentiable projective dynamics approaches have been used to optimize material distributions and actuation strategies [120], while differentiable design frameworks have been proposed for contact‐rich robotic structures and soft robot morphologies [121]. In addition, differentiable simulation has facilitated parameter identification and model refinement for soft materials, enabling learned corrections or calibrated physical parameters to be incorporated into simulation pipelines [116].

Despite these advantages, differentiable simulation also presents several limitations. Differentiating through complex physics solvers, especially those involving contact, discontinuities, stiff materials, or high‐resolution finite elements, can be computationally expensive and numerically fragile [122]. Many differentiable simulators rely on smooth approximations of contact or material behavior, which may introduce modeling inaccuracies in scenarios involving sharp impacts or highly nonlinear deformation [123]. Moreover, the requirement for differentiability can restrict the choice of numerical methods or necessitate reformulations that are less mature than their non‐differentiable counterparts. For soft robots with very high dimensional states or intricate material models, scalability remains an open challenge, and achieving stable, accurate gradients in these regimes is an active area of research.

#### 2.6.3 Practical Viability and Limitations

Differentiable simulation is most convincing for offline parameter identification, trajectory optimization, and design problems in which the material model and contact formulation remain sufficiently smooth. Its practical advantage becomes less clear when soft manipulation contains repeated contact creation, detachment, frictional switching, or high‐resolution finite‐element states. In these cases, gradients can be sensitive to contact smoothing, timestep selection, and solver convergence, while the backward calculation may approach the cost of repeated forward simulation. Differentiable simulation should therefore be viewed as a strong optimization tool under controlled modeling assumptions, rather than as a general solution for real‐time, contact‐rich soft‐robot dynamics [123].

#### 2.6.4 When Hybrid Intelligence May Fail

Hybrid intelligence does not guarantee that an inaccurate physical model and a flexible learner will form a reliable combined model. Its success depends on whether the analytical and learned components are assigned identifiable and compatible roles. Several failure conditions are particularly important for soft manipulators.

First, the physical backbone may contain the wrong structure. A residual learner can correct moderate errors in material parameters, actuation gain, damping, or local deformation response [102]. It is much less reliable when the nominal model omits a dominant mechanism, such as a contact mode, a hysteretic state, pressure‐flow dynamics, or a major geometric degree of freedom. In this case, the learned term must reproduce most of the system dynamics and the claimed physical interpretation becomes weak.

A useful diagnostic is the relative magnitude of the learned correction,

(28) $$\rho_{\mathrm{res}}=\frac{\left\|f_{\mathrm{learn}}\left(x,u\right)\right\|}{\left\|f_{\mathrm{phys}}\left(x,u\right)\right\|+\varepsilon },$$

where ε>0 avoids division by zero. This ratio is not a universal acceptance threshold, but it reveals how much of the prediction is being carried by the learned component. If $\rho_{\mathrm{res}}$ remains comparable to or greater than one over a large part of the operating range, the physical backbone or the selected state representation should be reconsidered.

Second, the decomposition may be non‐identifiable. The same measured error may be explained by a change in stiffness, an unmeasured hysteretic state, a contact force, or an arbitrary residual. Without sufficient sensing and excitation, these explanations cannot be separated from input–output data. The hybrid model may then predict the training trajectories accurately while assigning the error to the wrong component. This limits parameter interpretation and makes online adaptation unreliable.

Third, a smooth learned correction may fail at regime transitions. Contact onset, slip, detachment, valve saturation, and loading reversal can change the governing relations abruptly. A residual trained mainly in free motion may interpolate across the transition and produce incorrect force peaks or transition timing. Mode‐dependent models or explicit contact‐state estimation are needed when these transitions affect the task.

Fourth, the learned component may be asked to extrapolate beyond its data support. The physical model can provide a useful nominal trend, but it cannot guarantee that the residual remains bounded under unseen payloads, pressures, geometries, ageing, or contact surfaces. A hybrid model should therefore report the operating range used for calibration and should be tested with the learned component active and inactive under the same out‐of‐distribution conditions.

Finally, online adaptation can reduce rather than improve reliability. Rapid updates may fit sensor noise, temporary contact disturbances, or state‐estimation error. They may also alter damping, actuation gain, or feedback behavior faster than the controller can accommodate. Constrained parameter updates, uncertainty monitoring, dissipative conditions, or an independent safety layer are needed when the learned model is updated during operation [88].

Hybrid intelligence is therefore most viable when the physical model captures the dominant structure, the remaining discrepancy is smaller and better localized than the original dynamics, the relevant states are sufficiently observable, and the learned component is validated over the intended operating range. It may fail when weak physics and limited data are combined without identifiability, uncertainty bounds, or control‐level constraints. Under those conditions, hybridization adds complexity without providing a clear gain in physical credibility, generalization, or safety.

### 2.7 Spectrum: Physics‐Based to Data‐Driven

The methods discussed above should not be treated as isolated modeling choices. In soft manipulator research, they are better understood as points on a broader modeling spectrum that extends from fully analytical descriptions to highly flexible data‐driven representations [67, 124]. At one end of the spectrum are first‐principles continuum and finite‐element models, which derive system behavior from mechanics, geometry, material laws, and boundary conditions. At the other end are purely data‐driven models, which infer system dynamics directly from observations with minimal prior assumptions. Between these two ends sit many intermediate forms, including reduced‐order models, sparse equation discovery, operator‐theoretic lifting, continuous‐time neural dynamics, physics‐informed learning, neural operators, graph‐based learned simulators, and hybrid residual formulations [67]. Therefore, the real challenge is not to make a final choice between physics and data, but to decide how much prior structure is needed for a given task and where learning should be introduced.

A model that is appropriate for offline design optimization may be too slow for real‐time control. A model that performs well for free‐space motion may become unreliable during contact‐rich manipulation. A formulation that gives good short‐horizon predictions may still be unsuitable for estimation, stability analysis, or safe deployment. These differences arise because modeling quality in soft robotics cannot be judged by prediction accuracy alone. It also depends on computational cost, interpretability, robustness to regime changes, compatibility with sensing, and the extent to which the model can support control and decision‐making [67].

To make these distinctions explicit, Table 1 summarizes the representative modeling paradigms discussed in this section in terms of data efficiency, physical interpretability, computational cost, control readiness, online adaptability, and soft‐robot‐specific limitations. Explicit cross‐references to the corresponding sections are included in the table so that the basis for each qualitative assessment can be traced to the detailed discussion and supporting literature. The table is intended as task‐dependent qualitative guidance rather than as a universal ranking of modelling methods.

**TABLE 1:** Comparison of representative modeling paradigms for soft manipulators. ✓ indicates favourable, **▵** indicates conditional, and ✗ indicates limiting.

Paradigm	Data Eff.	Phys. Interp.	Low Cost	Control Ready	Online Adapt.	Main limitation in soft manipulators
Continuum / FEM (Sections 1 and 3.1)	✓	✓	✗	**▵**	✗	Mechanically reliable for design and validation, but direct online use becomes impractical for fine meshes, uncertain constitutive parameters, and repeated frictional contact [125, 126, 127, 128]
SINDy (Section 2.1)	**▵**	✓	✓	✓	**▵**	Viable for low‐dimensional and regime‐specific dynamics when history variables are represented. A single sparse model is unreliable across hysteresis loops and contact transitions [61, 129, 130, 131]
Koopman learning (Section 2.2)	**▵**	**▵**	**▵**	✓	**▵**	Strong for short‐horizon control within the sampled operating range. A global finite‐dimensional lifting may become excessively large or fail across changing contact modes and payloads [73, 80, 132, 133, 134]
Neural ODEs (Section 2.3)	**▵**	**▵**	**▵**	**▵**	**▵**	Useful for smooth continuous‐time and latent dynamics. Unconstrained models can become costly or unstable for stiff pneumatic response, long‐horizon rollout, and nonsmooth contact [103, 135, 136, 137]
PINNs (Section 2.4)	✓	✓	✗	**▵**	**▵**	Useful for smooth inverse and reconstruction problems with credible physics. Competing losses and imperfect equations can make full contact‐rich soft‐robot models difficult to train and validate [138, 139, 140, 141].
Neural operators / GNN simulators (Section 2.5)	✗	**▵**	**▵**	**▵**	**▵**	Efficient after training, but usually requires broad field‐level data and may generalise poorly across geometries or contact regimes [91, 135, 142, 143]
Hybrid physics–ML (Section 2.6)	✓	✓	**▵**	✓	✓	Effective when the physical model captures the dominant structure and the learned discrepancy remains bounded and identifiable. It can fail when the residual replaces most of the physics or adapts outside its validated range [144, 145, 146, 147, 148, 149, 150]

*Note*: The section references in the first column indicate where each modeling paradigm and the basis for its qualitative assessment are discussed in detail. The indicators summarize task‐dependent characteristics discussed in the corresponding sections and cited literature and should not be interpreted as universal benchmark scores. Data Eff., data efficiency; Phys. Interp., physical interpretability; Online Adapt., online adaptability.

From this viewpoint, the spectrum from physics‐based to data‐driven modeling is not simply a scale from older to newer methods. Moving along this spectrum does not mean replacing one paradigm with another. Rather, it reflects a shift in emphasis between fidelity, efficiency, interpretability, and deployability, depending on the modeling objective. On the more physics‐explicit side, Huang et al. used an absolute nodal coordinate formulation (ANCF)‐based kinematic framework for variable‐curvature soft continuum robots and reported computation times below 1 ms together with a mean end‐effector positioning error of 2.89% of total robot length [151]. This suggests that continuum models can remain physically grounded while still being fast enough for control‐oriented use. Closer to the reduced‐order middle of the spectrum, Sharma et al. used Lagrangian Operator Inference to compress a high‐fidelity soft‐robot model from 231 336 degrees of freedom to as low as 10 while keeping the extrapolation error below 2% in their reported test cases [152]. At the more compressed and deoyment‐oriented end, Tang et al. demonstrated that a learning‐based surrogate controller could achieve force‐position tracking errors substantially lower than probabilistic inference for learning control (PILCO) in contact‐rich manipulation, while Han et al. showed that Koopman lifting could improve both modeling and prediction accuracy relative to state‐space baselines in 2D and 3D soft robots [80, 105]. These examples illustrate that the choice between a more physical or more data‐oriented model often depends primarily on the actual task and deployment requirements, rather than on adherence to a fixed modeling paradigm.

A Neural ODE may be trained from data, but its latent state can still be chosen to reflect mechanical variables. A PINN may use neural approximation, but its solution space is constrained by governing equations. A hybrid model may begin from continuum mechanics, yet rely on learned residuals to remain accurate in practice [47, 102, 112, 124]. Even data‐driven methods like SINDy and Koopman do not involve unrestrained fitting; their core goal is to find a concise and structured representation of system behavior. Therefore, the real distinction is not whether the model learns, but how existing physical or prior knowledge is incorporated into the model. This prior knowledge can be reflected in the model representation, loss function, parameterization, or deployment strategy.

At the same time, the modeling spectrum should not be understood as a simple progression toward greater flexibility. Moving toward the data‐driven end often increases adaptability, but may reduce transparency and make extrapolation less reliable. Moving toward the physics‐driven end improves interpretability and consistency, but may increase computational burden and sensitivity to modeling assumptions. In soft robotics, neither side is universally optimal. The most suitable approach depends on the amount of available data, the quality of prior knowledge, the operating environment, and the model's intended use [67]. Therefore, a single modeling paradigm cannot dominate the field. Instead, the diversity of soft manipulator tasks and morphologies calls for a multi‐level perspective that matches different model structures with different modeling objectives.

The main implication is that soft manipulator modeling should be approached as a problem of model selection and model coordination, rather than as a search for a universally best representation. The question is not only how to improve any one model, but also how to decide which class of model is appropriate for a particular role, and how multiple classes of models can work together. This shifts the discussion from individual methods to modeling ecosystems. Once the field is viewed in this way, the next step is no longer to compare isolated approaches in abstraction, but to ask how they can be organized within a broader framework that links task requirements, model fidelity, real‐time constraints, and adaptive updating.

## 3 Integrated Modeling Ecosystem

### 3.1 Task‐Oriented Model Choice

The first question in an integrated modeling ecosystem is not which model is the most refined, but which model is sufficient for a given task under a given computational budget. In soft robotics, this distinction matters because model quality always depends on the output of interest, the operating regime, and the time available for estimation or control. A model that is adequate for quasi‐static grasp shaping may fail in dynamic tracking, while a model that is accurate for offline design optimization may be too slow for real‐time use. The central issue is therefore not model supremacy, but model allocation. This basic idea is summarized in Figure 6A, where model classes are arranged along a cost–fidelity spectrum and matched to representative task classes, from workspace exploration and kinematic planning to dynamic control and offline stress analysis.

**FIGURE 6:**
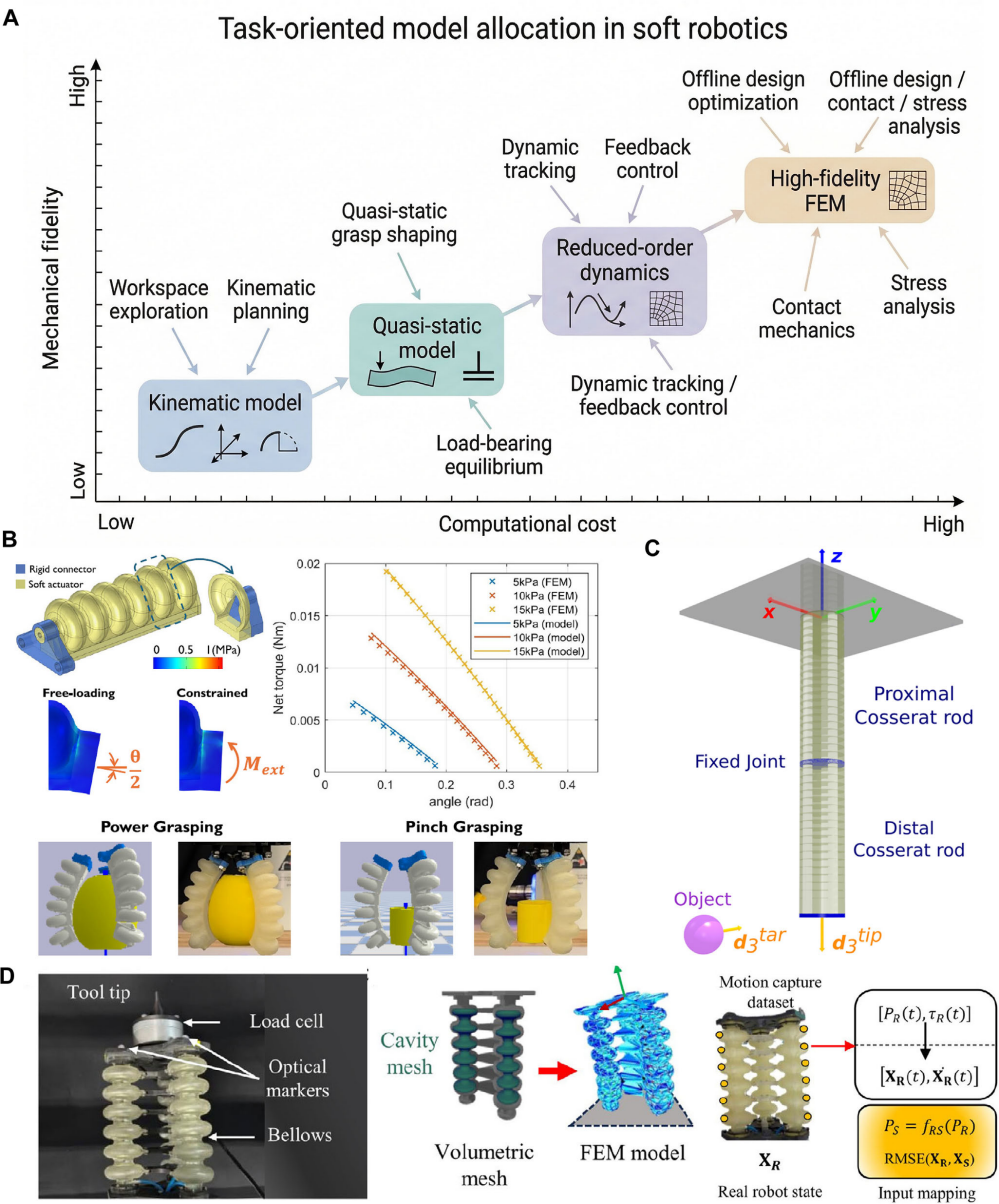
Task‐oriented model allocation in soft robotics. (A) Task‐dependent mapping between model classes and the cost–fidelity spectrum, from kinematic and quasi‐static models to reduced‐order dynamics and high‐fidelity FEM. (B) Validation of an FEM‐derived surrogate model for a soft gripper, including actuator‐level FEM modeling, torque–deflection agreement between the FEM and surrogate models, and simulation–experiment comparisons for power and pinch grasping. Reproduced with permission under the terms of the Creative Commons CC BY license 4.0 [153]. Copyright 2026, Wiley‐VCH. (C) Dynamic Cosserat‐rod simulation environment for a two‐module soft manipulator interacting with a target object in a pushing task. Reproduced with permission under the terms of the Creative Commons CC BY license 4.0 [154]. Copyright 2024, Wiley‐VCH. (D) Calibration workflow linking a physical pneumatic soft manipulator, a SOFA FEM model, motion‐capture state data, and pressure‐input mapping between the real and simulated domains. Adapted with permission under the terms of the Creative Commons CC BY license 4.0 [91]. Copyright 2026, Wiley‐VCH.

Therefore, model selection should be judged in relation to the task at hand. What matters is not whether a model is accurate in some abstract or universal sense, but whether it can predict the output that truly matters for the task with sufficient accuracy at an acceptable computational cost. Let $y_{T}$ denote the task‐relevant output of the real system and ${\hat{y}}_{T}$ the corresponding prediction produced by the model. The discrepancy between the two can then be written as

(29) $$E_{T}={\left\|{\hat{y}}_{T}-y_{T}\right\|}_{W_{T}},$$

where $W_{T}$ emphasizes which error components matter most for the task. From this perspective, the preferred model is the one with the lowest cost while still meeting the task error requirement. Here, cost should be interpreted broadly. It includes not only forward evaluation time, but also numerical robustness, memory demand, sensitivity availability, and worst‐case latency when the model is deployed in closed loop.

This view clarifies why different tasks lead to different model choices. In kinematic planning or coarse workspace exploration, the relevant outputs are usually backbone shape, end‐effector pose, or reachable‐set geometry. It is often sufficient to use constant‐curvature, piecewise‐constant‐strain, or related geometric representations, because inertial and transient effects do not strongly affect the planning layer. For motion generation in free space, this class of models often satisfies the task tolerance at very low computational cost. This is the low‐cost, lower‐fidelity corner shown in Figure 6A, where kinematic models are linked mainly to workspace exploration and kinematic planning rather than to load‐bearing or dynamic tasks.

The situation changes in quasi‐static grasp shaping or load‐bearing manipulation. Here the relevant output is no longer just pose, but the equilibrium configuration under external loading. A quasi‐static mechanics model is more appropriate,

(30) $$r(q,u,f_{\mathrm{ext}},\pi )=0,$$

where q denotes generalized deformation coordinates, u the actuation input, $f_{\mathrm{ext}}$ the external load, and π the material and geometric parameters. In this setting, the main issue is not inertial fidelity, but the ability to resolve compliance, load transfer, and contact‐induced deformation. For grasp synthesis or static manipulation maps, this class of models often provides a better balance than purely geometric approximations. The transition from kinematic to quasi‐static modeling is also explicit in Figure 6A, where quasi‐static models occupy the intermediate region needed for grasp shaping and load‐bearing equilibrium.

Dynamic tracking and control place a stricter requirement on temporal fidelity. In that case, a reduced‐order dynamic model becomes necessary,

(31) $$\begin{array}{ccc} & & M\left(q,\pi \right)\ddot{q}+C\left(q,\dot{q},\pi \right)\dot{q}+K\left(q,\pi \right)+D\left(q,\dot{q},\pi \right)\\ & & \;=B\left(q,\pi \right)u+J_{c}{\left(q\right)}^{⊤}\lambda , \end{array}$$

where M is the generalized inertia, C collects velocity‐dependent terms, K represents elastic restoring effects, D describes damping or dissipation, and $J_{c}^{⊤}\lambda$ denotes generalized contact forces when applicable. This class is especially relevant when the task depends on phase lag, transient overshoot, or input‐rate effects. The value of Cosserat‐based dynamic reduction is precisely that it preserves the main mechanical structure while remaining compatible with real‐time execution [18]. Figure 6C illustrates this control‐oriented role using a dynamic Cosserat‐rod model of a two‐module soft manipulator interacting with a target object. The reduced mechanical representation retains the geometry and contact dynamics needed for learning‐based control without resolving the full continuum field.

At the highest end of fidelity, one uses a spatially resolved model, often finite‐element based. In semi‐discrete form, the governing equation can be written as

(32) $$M\left(d\right)\ddot{d}+f_{\mathrm{int}}\left(d,\dot{d}\right)=f_{\mathrm{ext}}\left(u,d\right)+f_{c},$$

where $d\in R^{N}$ denotes nodal degrees of freedom. This class is well suited to offline design optimization, contact‐rich planning benchmarks, or surgical rehearsal, where computation can be amortized offline and fidelity matters more than servo‐rate execution [20]. Figure 6B illustrates a related fidelity–efficiency strategy. High‐fidelity FEM is used to characterize actuator behavior, while a compact surrogate reproduces the torque–deflection response for task‐level grasping simulation and is subsequently compared with physical grasping experiments.

The ecosystem view matters because no single model class dominates across all of these tasks. In practice, model choice is better understood as an operating point on an accuracy–cost frontier than as a universal optimum. This is also why internal resolution matters within each class. The practical fidelity of a model is shaped not only by whether it is kinematic, quasi‐static, reduced‐order, or high‐fidelity, but also by how many sections, modes, reduced coordinates, or mesh elements it uses.

Real‐time Cosserat dynamics show that dynamic structure can be preserved without resorting to full high‐dimensional simulation [18]. Real‐time FEM remains an important baseline when high‐fidelity mechanics are needed inside a control loop, but it also makes clear that such fidelity comes at a computational cost [20]. Work on soft parallel robots further shows that different levels of model fidelity become useful at different stages, from kinematic characterization to dynamic control [112, 155]. Figure 6D provides a complementary calibration example, linking a physical pneumatic manipulator with a SOFA FEM model through motion‐capture state measurements and pressure‐input mapping. This illustrates how a high‐fidelity model can be aligned with hardware before reduced or learned models are introduced for faster online use.

### 3.2 Complementary, Not Competing

Section 2.6 discussed hybrid physics–machine learning at the level of model construction. Here we shift the focus from the model itself to the system architecture in which the model is used. In soft manipulation, physics‐based and learning‐based models are most useful when they are assigned different operational roles. The physics‐based component provides coordinate meaning, mechanical consistency, nominal extrapolation, and safety‐relevant constraints. The learned component provides compression, residual correction, parameter adaptation, or fast prediction where the physical model is incomplete or too slow.

This complementary view is illustrated at several levels in Figure 7. Figure 7A summarizes the overall architecture, linking physics‐based modeling, learned correction, state estimation, parameter updating, predictive control, and hardware feedback. Panels B–D provide representative examples of these roles in practice, including reduced model‐based parameter identification and control, learning‐based actuation, and a hardware‐synchronized digital‐twin framework.

**FIGURE 7:**
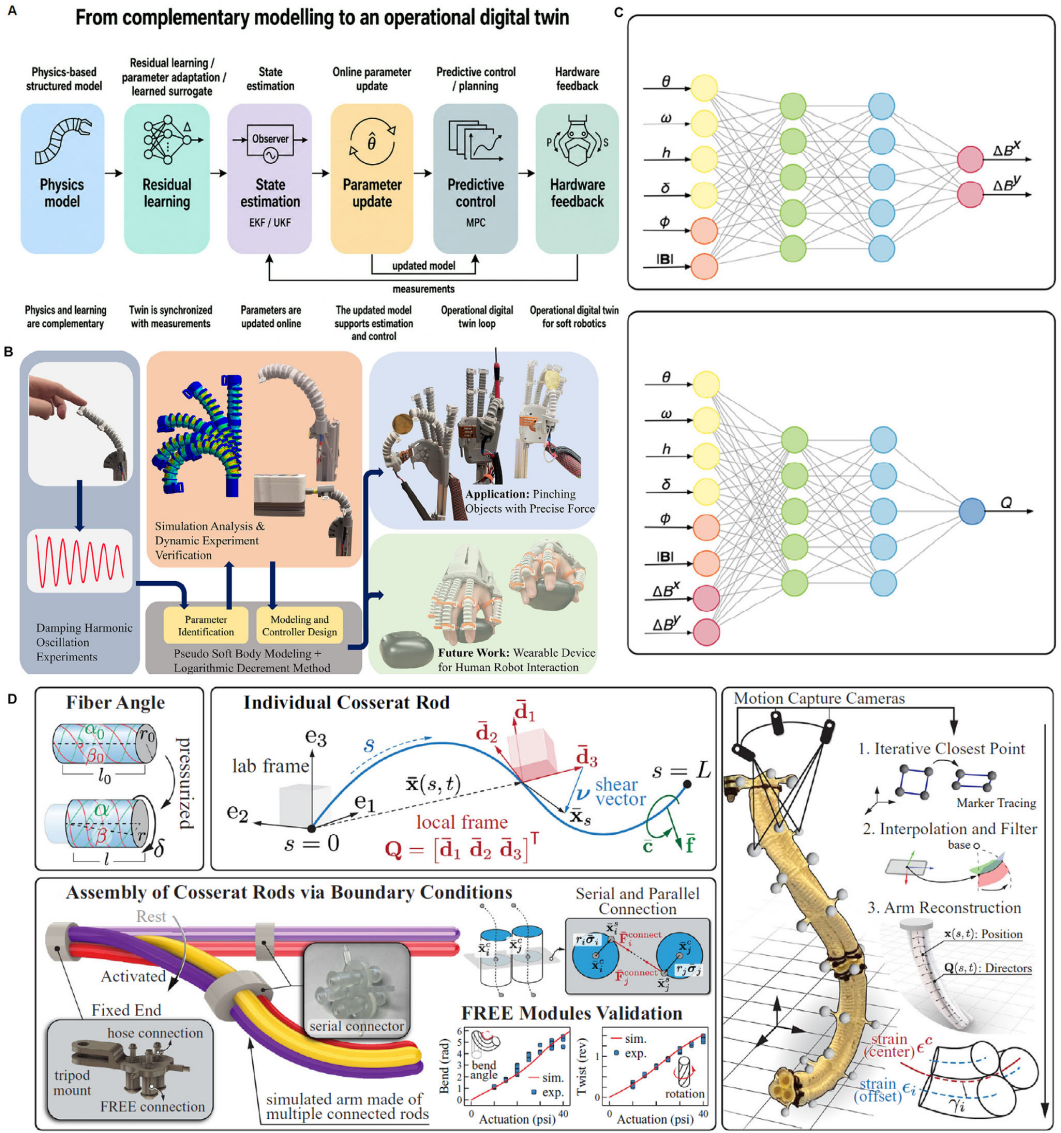
Complementary modeling, learning, and digital‐twin components for soft robotic systems. (A) Closed‐loop workflow linking a structured physics model, learned correction, state estimation, online parameter update, predictive control, and hardware feedback. (B) Model‐based soft‐hand control workflow combining damped‐oscillation experiments, logarithmic‐decrement parameter identification, pseudo‐rigid‐body modeling, and position/force control. Reproduced with permission under the terms of the Creative Commons CC BY license 4.0 [156]. Copyright 2025, Wiley‐VCH. (C) Actor–critic networks for deep‐reinforcement‐learning‐based adaptive actuation of magnetic soft robots, where the learned policy maps robot and magnetic‐field states to actuation commands. Adapted with permission under the terms of the Creative Commons CC BY license 4.0 [157]. Copyright 2023, Wiley‐VCH. (D) Digital‐twin framework for modular fiber‐reinforced soft continuum arms, combining Cosserat‐rod assembly models, simulation validation, motion‐capture reconstruction, and physical hardware. Adapted with permission under the terms of the Creative Commons CC BY license 4.0 [158]. Copyright 2026, Wiley‐VCH.

A compact discrete‐time representation of this complementary architecture is

(33) $$x_{k+1}=\Phi_{\mathrm{phys}}\left(x_{k},u_{k};{\hat{\pi }}_{k}\right)+r_{\theta }\left(x_{k},u_{k}\right),$$

where $\Phi_{\mathrm{phys}}$ denotes the discrete‐time transition associated with the calibrated physical model, ${\hat{\pi }}_{k}$ denotes the current estimate of the physical parameters, and $r_{\theta }$ denotes a learned residual correction. The residual is therefore introduced only after the physical model has accounted for the dominant known dynamics. Unlike the model‐selection discussion in Section 2.6, the emphasis here is not on where the residual should be placed, but on how the residual model is connected to sensing, updating, and control.

For control deployment, the complementary architecture must also preserve the safety and stability role of the nominal model. The learned correction should therefore remain bounded, monitored, or filtered so that it does not invalidate pressure limits, contact‐force limits, workspace constraints, or closed‐loop damping. This requirement connects complementary modeling directly to digital‐twin architectures: the model is continuously synchronized with hardware, updated only within a validated range, and used for decision support under explicit computational and safety constraints.

### 3.3 Toward a Digital Twin

The integrated modeling ecosystem described above becomes a digital twin when it maintains a persistent and measurement‐updated representation of a specific physical robot. A digital twin is therefore more than a simulator. Its defining feature is the persistent synchronization between the physical system and its digital representation through measurements, allowing the updated model to support monitoring, prediction, planning, or control [46]. Figure 7A provides a concise schematic of this transition, showing how state estimation, parameter adaptation, learned correction, predictive control, and hardware feedback can be integrated within such an operational architecture. Recent soft‐robotic frameworks have demonstrated related mechanisms through online state estimation, model updating, adaptive control, and physical–virtual synchronization [111, 159, 160, 161].

Within the terminology of this Perspective, a digital twin is one possible operational realization of hybrid intelligence, but the two terms are not interchangeable. Hybrid intelligence describes the architecture‐level coordination of physics‐based models, learned components, state estimation, adaptation, and task‐level decision‐making. A digital twin, by contrast, additionally requires a persistent digital representation associated with a specific physical system and updated through measurement‐driven synchronization [46]. A digital twin may rely predominantly on a physics‐based model and therefore does not necessarily constitute hybrid intelligence. Conversely, a hybrid‐intelligence framework may support offline co‐design, model coordination, or adaptive control without maintaining a continuously synchronized digital representation. The two concepts overlap when physics‐based and learned components are coordinated within a measurement‐updated representation that directly supports prediction and decision‐making for the physical robot [102, 111, 159].

For soft robots, the physical state is effectively distributed. Let χ(s,t) denote a continuum description along the arm‐length coordinate s∈[0,L], including quantities such as backbone pose, strain, and pressure distribution. A practical digital twin requires a finite‐dimensional representation of this distributed state. We write

(34) $$x={\left[\begin{array}{ccc} q^{⊤} & {\dot{q}}^{⊤} & z^{⊤} \end{array}\right]}^{⊤},$$

where q collects reduced configuration coordinates, $\dot{q}$ denotes their rates, and z represents latent dissipative, hysteretic, or other unresolved dynamic states. This distinction allows the digital twin to estimate dynamic states and physical parameters through different update mechanisms.

The first layer of a digital twin is state synchronization. In discrete time, one may write

(35) $$\begin{array}{cc} x_{k+1} & =f\left(x_{k},u_{k},\pi_{k}\right)+w_{k}, \end{array}$$

(36) $$\begin{array}{cc} y_{k} & =h\left(x_{k}\right)+v_{k}, \end{array}$$

where $u_{k}$ is the actuation input, $y_{k}$ the measurement vector, and $w_{k},v_{k}$ effective process and measurement noise. For moderate nonlinearity, this synchronization layer can be implemented through an extended or unscented Kalman filter. Because measurements in soft robots are sparse, the estimator is not merely denoising sensor data, but regularizing an ill‐posed inverse problem with the model acting as a strong prior. The quality of synchronization is therefore inseparable from model quality. Figure 7D provides a hardware‐synchronized example, where motion‐capture measurements are used to reconstruct the configuration of a modular soft continuum arm and validate its Cosserat‐based digital representation against the physical system.

The second layer is parameter adaptation. This is necessary because a synchronized state estimate alone is often not enough when the robot softens, stiffens, drifts, and interacts with a changing environment. This layer can be implemented either by augmenting the estimator state with slowly varying parameters or by solving a short‐horizon online fitting problem over recent measurements. In both cases, adaptation allows the model to evolve in tandem with the hardware, rather than remaining frozen at its calibration condition. This role is already visible in Figure 7A, where parameter updating is placed between state estimation and predictive control, emphasizing that model adaptation can form part of the operational architecture rather than an occasional offline recalibration.

The third layer is decision support. The synchronized and adapted twin should not remain only an observer. It should provide the model used by planning and control. In a predictive control setting, one solves

(37) $$\begin{array}{ccc} & & \min_{u_{0:N-1}}\sum_{j=0}^{N-1}\ell \left(x_{j},u_{j}\right)+\ell_{f}\left(x_{N}\right)\\ & & \text{s.t.}\;x_{j+1}=f_{\mathrm{twin}}\left(x_{j},u_{j},{\hat{\pi }}_{k}\right),\;x_{0}={\hat{x}}_{k}, \end{array}$$

where ${\hat{x}}_{k}$ and ${\hat{\pi }}_{k}$ are the current synchronized state and adapted parameters. At this point, the controller no longer uses a fixed nominal model, but instead uses the current model instance maintained by the twin.

This three‐layer interpretation gives a definition of what a soft‐robot digital twin should do. It should synchronize hidden states from sparse sensors, adapt slowly varying constitutive and actuation parameters, and then provide predictive support for planning or control. Recent work fits naturally into this structure. A multi‐fingered soft gripper digital twin has been proposed as a platform for underactuated control and uncertainty‐aware learning, showing that the twin can serve as a computational environment for decision‐making rather than just visualization [160]. A digital twin framework for modular fiber‐reinforced soft continuum arms combines Cosserat‐based modeling, three‐dimensional reconstruction, and interactive control design, which directly supports the idea that design, estimation, and deployment should share a common model substrate [158]. Adaptive MPC with a PINN surrogate based on Cosserat rod theory makes the connection even more concrete: the same surrogate model is used for state estimation, compliance adaptation, and control, which is close to an operational definition of a digital twin [159]. Recent work on simultaneous state estimation and online model learning further reinforces the second layer, showing that pose estimation and online stiffness learning can be handled together rather than as separate stages [111].

A central challenge for soft‐robot digital twins is the sim‐to‐real gap. This challenge is especially severe for soft manipulators because their behavior is highly sensitive to fabrication variability, uncertain material parameters, viscoelastic hysteresis, pneumatic delay, frictional contact, sensor calibration errors, payload changes, and long‐term material ageing [102, 162, 163, 164]. As a result, a digital twin calibrated under one operating condition may lose predictive validity when the robot enters contact‐rich or load‐varying environments [102, 111, 159, 163]. The role of the twin is therefore not to eliminate model–plant mismatch completely, but to make this mismatch observable, bounded, and correctable during operation.

Several strategies can mitigate this gap. Domain randomization can expose the model or controller to distributions of stiffness, damping, geometry, friction, contact conditions, actuation delay, payload, and sensing noise before deployment, thereby reducing its sensitivity to a single nominal simulator [165, 166]. Rather than selecting these distributions manually, adaptive domain‐randomization approaches can refine the simulator‐parameter distributions using real‐world trajectories or informative simulated conditions [167, 168, 169, 170]. System identification and hardware calibration can then narrow these distributions for a specific robot by identifying material, damping, geometric, and actuation parameters from experimental measurements [162, 163].

During operation, residual learning can correct systematic discrepancies between simulated and measured behavior [102]. Following the residual definition in Equation (33), the learned correction $r_{\theta }$ in the complementary architecture can be trained to predict the repeatable component of the model–plant mismatch. In a digital‐twin setting, this correction is updated using hardware measurements and applied together with the current state and parameter estimates. The resulting model therefore retains the structure of the calibrated simulator while accounting for discrepancies caused by hysteresis, pneumatic delay, contact, fabrication variability, and material drift. The residual therefore represents the model–plant mismatch that remains after state synchronization and parameter adaptation. This residual correction can be combined with online state and parameter estimation, such as an Extended Kalman Filter (EKF), an Unscented Kalman Filter (UKF), or moving‐horizon estimation, so that slowly varying quantities such as stiffness, damping, pressure‐to‐force gain, and contact‐dependent parameters can be updated from hardware measurements [111, 159, 171, 172]. For control, uncertainty‐aware or robust model predictive control can prevent the controller from over‐trusting the twin when prediction confidence is low and can explicitly account for bounded or probabilistic model uncertainty [159, 173, 174]. In this sense, a soft‐robot digital twin should be understood as an adaptive sim‐to‐real interface: it uses simulation to retain physical structure, real measurements to maintain synchronization, and learned correction to account for the mismatch that remains.

Digital twins should not be viewed as a rebranding of simulation technology. In soft robotics, they are the natural endpoint of the integrated modeling ecosystem. Task‐oriented model choice determines which model class is feasible. Complementary modeling determines how physical structure and learned corrections are combined. The digital twin then embeds these choices into a recursive loop of synchronization, adaptation, and decision support.

## 4 Research Implications

### 4.1 Real‐Time Control Feasibility

A central implication of the modeling shift reviewed in this Perspective is that the value of a model for soft manipulators increasingly depends on its ability to support control at the timescale required by the task. In this context, real‐time feasibility should not be interpreted merely as rapid forward prediction. Rather, a control‐oriented model must support reliable state estimation, predictable computational latency, and robust performance under changing operating conditions, as highlighted in recent surveys of soft‐robot modeling and control [16, 114]. This requirement is especially demanding in soft manipulation because the same system may transition rapidly between free motion, contact formation, sliding, and load‐bearing interaction, with each regime altering the effective dynamics relevant to control [16].

This perspective clarifies why high‐fidelity continuum and finite‐element models, although indispensable for mechanistic understanding and design validation, are often difficult to deploy directly within closed‐loop control architectures. Their limitations arise less from a lack of physical relevance than from their computational burden, parameter sensitivity, and reduced tractability under contact‐rich conditions, all of which are well documented in nonlinear finite‐element theory and recent reviews of soft‐robot simulation [67, 175]. By contrast, reduced‐order and structure‐preserving models occupy a more practical position for control. As discussed earlier in this paper, approaches such as SINDy [34], Koopman lifting [71], and reduced Cosserat formulations [18] compress nonlinear soft‐robot behavior into model forms that remain compatible with feedback design while avoiding the full cost of continuum simulation. The quantitative results compiled in Table 2 make this trade‐off more explicit, spanning low‐order kinematic models with sub‐millisecond simulation time [151] and reduced‐order finite‐element or operator‐based formulations designed for control‐oriented deployment [68, 152, 176].

**TABLE 2:** Representative quantitative results relevant to real‐time control of soft manipulators. The reported values are collected from heterogeneous platforms, tasks, sensing conditions, and evaluation protocols, and should therefore be interpreted as representative performance scales rather than direct comparisons.

Method	Task	Platform / condition	Model scale	Runtime / update rate	Reported result	Interpretation
Variable‐curvature kinematic modeling and control [151]^a^	Continuum robot tracking	Two‐section pneumatic soft continuum robot	Low‐order	<1 ms	Mean end‐effector error of 2.89% of robot length	Shows real‐time value for low‐order shape tracking.
Reduced‐order finite‐element optimal control [68]^b^	Cable‐driven robot control	Cable‐driven “Diamond” soft robot	9768‐DOF FEM → 42‐state ROM	Mean QP time 17 ms	End‐effector tracking MSE of 0.07 ${\mathrm{mm}}^{2}$	Shows how FEM reduction can enable real‐time optimal control.
Offset‐free Koopman model predictive control [176]^c^	Trajectory tracking	Pneumatic and cable‐driven soft manipulators	Lifted linear model	MPC‐compatible	Tracking error reduced by 30.0% in free space and 45.4% in confined space	Shows improved tracking using offset‐free lifted prediction.
Lagrangian Operator Inference for structure‐preserving reduction [152]^d^	Control‐oriented surrogate modeling	High‐dimensional simulated soft swimming robot	231,336‐DOF FOM → low‐dimensional ROM	Reduced‐order	Improved predictive accuracy and robustness to unseen inputs	Shows scalable structure‐preserving model reduction.
Physics‐learning hybrid dynamic modeling and control [112]^e^	Dynamic trajectory tracking	Pneumatic soft parallel robot	Hybrid physics–learning model	<3 ms; 50 Hz control	Feedforward error reduced by 53%–67%; feedback error of 0.4–1.1 mm	Shows accuracy gains from physics–learning correction.
Koopman‐based control of inertial soft robots [82]^f^	Inertial soft robot control	Two morphologically different pneumatic soft arms	Data‐driven control model	60 Hz; <5 min training data	Closed‐loop tip speed up to 1.52 m/s and acceleration of 11.6 m/$s^{2}$	Shows rapid model construction for dynamic control.
Physics‐informed surrogate MPC [103]^g^	Articulated soft robot control	Five‐joint pneumatic articulated soft robot	Physics‐informed neural surrogate	Up to 467× faster; 46.3–47.7 Hz MPC	Mean joint‐tracking error of 2.29–2.98 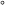	Shows real‐time MPC enabled by a fast surrogate.

*Notes*. DOF, degrees of freedom; FOM, full‐order model; MSE, mean squared error; NMPC, nonlinear model predictive control; QP, quadratic programming; ROM, reduced‐order model.

a Two‐section pneumatic continuum robot; fixed control frequency not reported; error is mean end‐effector position error normalized by robot length.

b Four‐cable Diamond robot; optimization at 20 Hz and feedback/state estimation at 100 Hz; error is Cartesian end‐effector MSE.

c Pneumatic and cable‐driven soft manipulators; fixed loop frequency not reported; values are relative reductions in trajectory‐tracking error against the baseline Koopman controller.

d Simulated six‐actuator soft swimmer; simulation step 1 ms; error is the relative Frobenius‐norm output error between full‐ and reduced‐order trajectories.

e Three‐channel pneumatic soft parallel robot; control at 50 Hz; error is end‐effector trajectory‐tracking error.

f Four‐muscle and three‐muscle pneumatic soft arms; data collected at 60 Hz; tracking is evaluated from tip‐position deviation from the reference.

g Five‐joint antagonistic‐pneumatic robot; NMPC at 46.3–47.7 Hz; error is the mean absolute joint‐position error averaged over five joints.

Real‐time feasibility, however, is not achieved by model reduction alone. Actuation‐side dynamics are equally important in determining the achievable control bandwidth. For example, fiber‐reinforced soft bending actuators have been controlled using high‐speed on/off valves together with an explicit pneumatic dynamic model, illustrating how valve dynamics and pressure regulation become part of the real‐time control problem [177]. A model that is sufficiently fast in free space may still fail once contact, hysteresis, fluidic delay, or payload variation become significant. For this reason, the most promising direction is not a strict choice between physics‐based and data‐driven control models, but their principled coordination. Complementary and hybrid formulations are particularly important because they preserve the dominant mechanical structure while allowing learned components to absorb persistent mismatch. In practical terms, this suggests that real‐time control for soft manipulators will rely less on either full first‐principles simulation or unconstrained black‐box prediction in isolation, and more on models that are deliberately incomplete yet systematically correctable. This trend is reflected in recent work on Koopman‐based control of inertial soft robots [82], physics‐learning hybrid dynamic control [112], and physics‐informed surrogate MPC [103], all of which are summarized in Table 2. Model‐based reinforcement learning also provides a relevant control‐oriented perspective. Early work on soft manipulators used a learned forward dynamics model inside a model‐based policy‐learning framework, showing that predictive control can be learned even when analytical dynamics are difficult to use directly [32]. More recent work by Jitosho et al. demonstrated reinforcement‐learning‐based real‐time planning and control of agile manoeuvres for soft robot arms, using simulation and actuator modeling to support transfer to hardware [178]. These studies are important for the present discussion because they treat the learned model not only as a predictor, but as a component inside a decision‐making and control architecture. They therefore fit naturally within the broader hybrid‐intelligence view developed in this Perspective.

This shift also changes how feasibility should be evaluated. For control deployment, model quality should be assessed not only by trajectory fit, but also by end‐to‐end control relevance, including worst‐case inference time, numerical reliability, compatibility with observers and MPC, robustness to regime changes, and tolerance to sensing sparsity. From this perspective, a useful benchmark for future soft‐manipulator modeling is not whether a given method is universally the most accurate, but whether a modeling stack can deliver stable, adaptive, and computationally bounded performance in closed loop. Within this broader view, digital‐twin‐style architectures become particularly significant because they offer a mechanism for synchronizing hidden states, updating uncertain parameters online, and maintaining a control‐ready predictive model during operation; this potential is already suggested by recent work on learned residual physics and soft‐robot digital twins [102, 158, 179].

Taken together, these results suggest that real‐time control feasibility is not a property of model speed alone. A model may be fast in free space yet fail under contact, payload variation, hysteresis, or sensing uncertainty. For soft manipulators, the more relevant question is therefore whether a modeling stack can remain computationally bounded and sufficiently reliable when the operating regime changes. This is precisely where complementary physics–learning strategies become important. A physics‐based backbone can preserve the dominant mechanical structure, while residual learning, online parameter adaptation, or lifted representations can absorb the mismatch that would otherwise degrade controller performance. In this sense, real‐time feasibility is best understood as an ecosystem property: offline high‐fidelity mechanics for validation and calibration, combined with reduced or hybrid online models for estimation, prediction, and control.

This also changes how modeling performance should be evaluated. For deployment‐oriented soft robotics, the key benchmark is not simply prediction accuracy in isolation, but whether a model supports stable, timely, and adaptive closed‐loop behavior. Metrics such as worst‐case inference time, solver convergence under receding‐horizon control, robustness to unseen inputs, tolerance to sensing sparsity, and resilience under contact‐rich interaction are therefore at least as important as nominal fit error. Real‐time control feasibility thus provides one of the clearest practical tests of the broader transition argued in this paper: the move from isolated modeling paradigms toward integrated, hybrid‐intelligence modeling ecosystems for soft manipulators.

### 4.2 Design Optimization Acceleration

For soft manipulators, the design problem is rarely only about finding a shape that bends well. Geometry, material stiffness, actuation, sensing, and control are usually linked together. For example, a larger chamber may give a wider bending range, but it may also need higher pressure or make the response slower. A softer finger may improve contact with an object, but it may also become harder to control under load. Because of this, a useful model should help the designer move faster from a task requirement to a design that can be fabricated, controlled, and tested on hardware. Manufacturing and material functionality can further enlarge this design space. Bioinspired soft crawlers make this interdependence particularly clear, because locomotion performance emerges from the combined selection of compliant materials, body mechanisms, and actuation strategies [180]. Recent 3D digital light printing of self‐healing and reprocessable soft robots shows that complex pneumatic geometry, recoverability, and material reprocessability can become explicit design considerations rather than fixed post‐fabrication properties [181, 182].

The main difficulty is that accurate models are often too slow for repeated design search. FEM and continuum models can describe deformation and contact in detail, but they are expensive when thousands of candidate designs or control inputs must be tested. Recent work therefore often uses a two‐level strategy. High‐fidelity simulation or experiments are used to provide reliable data, and a faster model is then built for repeated optimization. In this way, the model does not need to reproduce every detail of the soft body. It needs to keep the task‐relevant information, such as shape error, grasp strength, tracking error, pressure demand, or control frequency.

A clear example is the Soft Pneumatic Actuator Design frAmework (SPADA) toolbox proposed by Yao et al. for bellow soft pneumatic actuator design [183]. In this work, FEM simulations were used to generate training data, and an artificial neural network (ANN) surrogate was then used for fast shape‐matching design. The target curve was divided into piecewise constant‐curvature sections, and a surrogate‐assisted genetic algorithm searched for suitable actuator parameters. The designed actuators achieved 2D shape‐matching Root Mean Square Error (RMSE) values of 4.16, 2.70, and 2.51 mm in the reported cases. As shown in Figure 8A, this model helps convert a desired shape into a printable actuator design with less manual trial and error.

**FIGURE 8:**
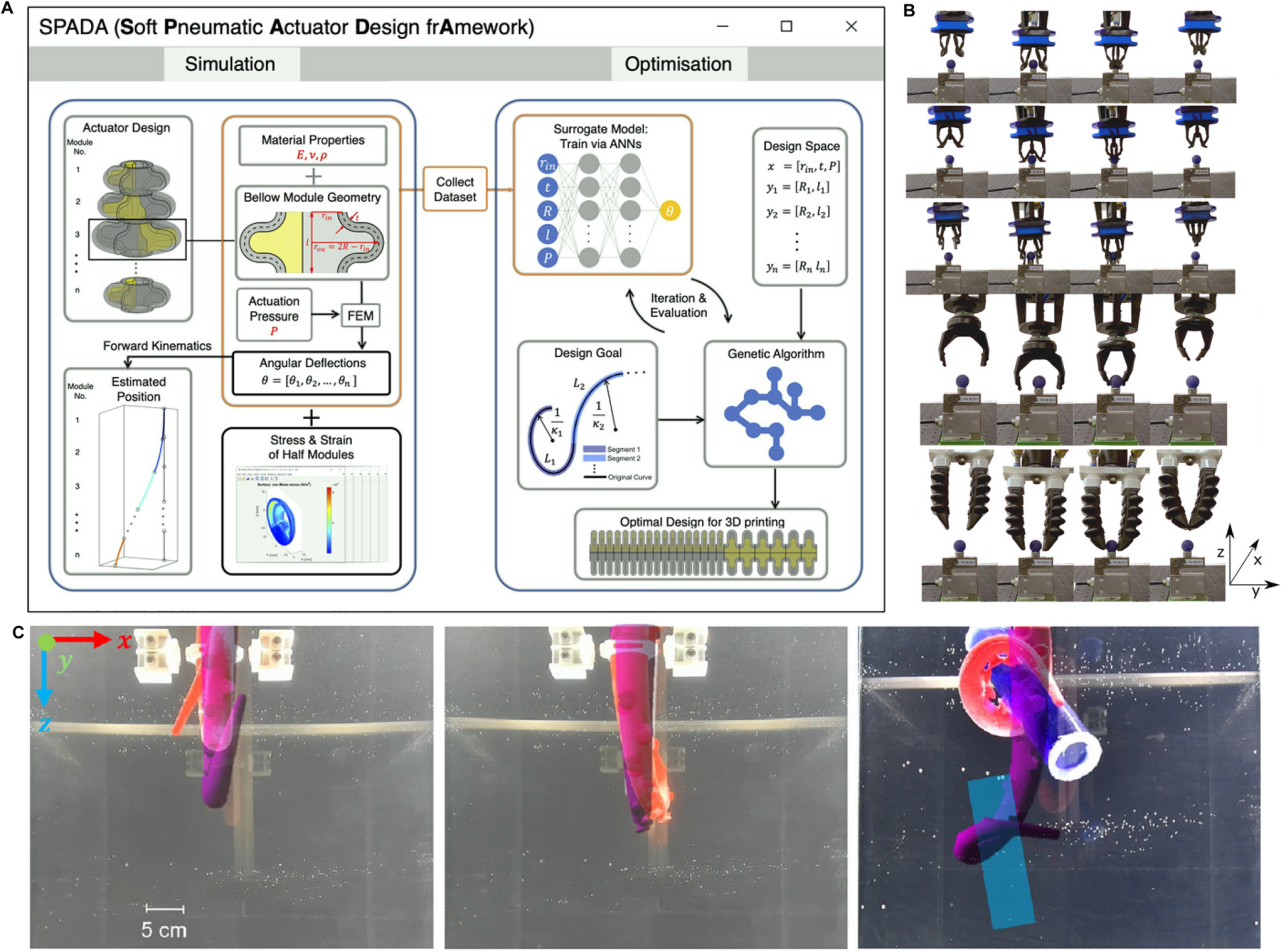
Model‐supported design acceleration in soft manipulation. (A) SPADA uses FEM‐generated data to train a fast ANN surrogate, allowing target shapes to be converted into manufacturable bellow soft pneumatic actuator designs through segmentation and genetic search. Reproduced with permission under the terms of the Creative Commons CC BY license 4.0 [183]. Copyright 2024, Sage Publishing. (B) Diversity‐based topology optimization of multi‐material soft grippers, followed by fabrication and large‐scale experimental grasp testing of optimized designs. Reproduced with permission under the terms of the Creative Commons CC BY license 4.0 [184]. Copyright 2024, Wiley‐VCH. (C) Experimental validation of optimized tendon routing for an octopus‐inspired cable‐driven soft manipulator, demonstrating designed underwater configurations on physical hardware. Adapted with permission under the terms of the Creative Commons CC BY license 4.0 [185]. Copyright 2026, Wiley‐VCH.

Design becomes more useful when it is considered together with control. Mannam et al. studied design and policy co‐optimization for dexterous anthropomorphic soft hands [186]. Their framework evaluated nearly 400 hand designs in simulation and then tested the optimized hands in more than 900 real teleoperated manipulation tasks. The optimized hands performed better than earlier manually designed versions in pick‐up and reorientation tasks.

Pinskier et al. developed a diversity‐based topology optimization method for multi‐material soft grippers [184]. The study combined computational design, simulation, fabrication, and automated grasp testing. The authors evaluated the optimized grippers over 15 170 real grasp tests, including tests of grasp strength, robustness, and durability. Some optimized candidates achieved higher grasp strength than comparable commercial grippers. As shown in Figure 8B, this example shows why hardware data are important in design optimization. Simulation can reduce the design space, but real task tests are still needed to identify which designs actually work. Figure 8C provides a complementary example at the continuum‐arm level. Martini et al. [185] combined a genetic algorithm with finite‐element modeling to optimize the number and routing of tendons in an octopus‐inspired soft manipulator. Selected designs were subsequently fabricated and validated underwater, providing a direct comparison between simulation‐based design optimization and physical realization.

Recent generative and differentiable methods further extend this direction. Song et al. proposed MorphVAE for voxel‐based soft robot morphology design [187]. The method uses a task‐conditioned variational autoencoder to generate soft robot morphologies and reuse design information across tasks. In six Evolution Gym tasks, MorphVAE showed faster search and higher fitness than several baseline methods in most cases. Although this work is mainly simulation‐based, it shows how generative models can reduce the cost of exploring large morphology spaces. For magnetic soft robots, Wang et al. combined topology optimization with a magneto‐elastic material point method to co‐design structure, local magnetization, and time‐varying magnetic actuation under large deformation, dynamic motion, and contact [188]. This type of work suggests that soft robot design is moving from simple parameter tuning to joint optimization of morphology, material distribution, and actuation.

The same idea also matters after the design is chosen. A soft robot that is well designed but too slow to model is still hard to control in real time. Licher et al. used a domain‐decoupled physics‐informed neural network as a surrogate for a dynamic Cosserat rod model in adaptive MPC for soft continuum robots [159]. The surrogate speed‐up was about 44,000 times, and the controller ran at 70 Hz. The end‐effector error was below 3 mm in simulation, with similar accuracy in real experiments. This example connects model acceleration directly with control. A fast model can be used inside the controller, rather than only for offline analysis.

These studies show that design optimization acceleration is not just about generating more candidate robots. The more important goal is to reduce the number of slow manual cycles between a task idea, a manufacturable design, a working controller, and hardware validation. Surrogate models make design search faster. Co‐design methods connect morphology with control. Automated testing gives task‐level data. Real‐time surrogates then allow the selected design to be controlled on hardware. Together, these steps make the modeling process closer to real deployment.

### 4.3 Adaptive Environmental Learning

Adaptive environmental learning refers to the ability of a soft manipulator to update its model or control strategy in response to changing task and environmental conditions. This capability is particularly important because soft robotic behavior is strongly shaped by external interaction, as discussed in model‐based control surveys of soft robots [16] and in learning‐based studies of soft robot–environment interaction [105]. Contact location, surface geometry, friction, payload variation, actuation history, and material ageing can all alter the relationship between input and deformation. As a result, a model identified under one operating condition may not remain valid when the robot is deployed in a new environment.

In conventional modeling workflows, environmental variation is often treated as a disturbance or as an uncertainty to be minimised during calibration. For soft manipulators, however, this view is often too limited. Environmental interaction, as shown in force and position tracking studies of soft robot–environment interaction [105], is not an exceptional case but a central part of soft manipulation. The same compliance that enables safe contact also makes the system sensitive to changes in load path, boundary condition, and contact state. Therefore, future modeling frameworks must move beyond static offline identification and toward adaptive models that can be refined during operation, including online state‐estimation and model‐learning frameworks such as that proposed by Ewering et al. [111].

From a hybrid‐intelligence perspective, adaptive environmental learning should not be understood as unconstrained online learning. A more useful formulation is to keep the mechanically meaningful structure of the model explicit while updating only the uncertain or environment‐dependent components. This idea is consistent with learned residual physics for soft‐robot sim‐to‐real transfer [102], physics‐informed adaptive model‐predictive control based on Cosserat rod theory [159], and online model‐learning approaches for soft robotic systems [111]. For example, a physics‐based or reduced‐order model may describe the nominal deformation and actuation response, while a learned residual term accounts for contact‐induced errors, pressure‐flow mismatch, frictional effects, or payload‐dependent changes. In this way, adaptation is used to correct the model where the real uncertainty lies, rather than to relearn the entire system from data. Model‐based contact detection and accommodation provides a complementary example of this principle: contact‐induced changes in a fiber‐reinforced soft bending actuator can be detected from its dynamic response and incorporated into an adaptive robust control framework rather than requiring the entire model to be relearned [189].

This adaptive capability also depends on sensing. Soft sensing technologies are therefore not only peripheral hardware, but a necessary condition for adaptive modeling. Recent reviews have summarized a wide range of sensing modalities for soft robots, including resistive, capacitive, optical, magnetic, piezoelectric, triboelectric, pneumatic, tactile, and vision‐based sensing [190, 191]. Integrated actuation–sensing designs further show that the boundary between actuator, sensor, and body can become blurred in soft robots, allowing the same compliant structure to generate motion and provide proprioceptive or haptic feedback [192]. Advanced piezoelectric materials extend this integration further by connecting mechanical deformation with sensing, actuation, and energy harvesting, offering a route toward more functionally integrated and potentially self‐powered soft robotic systems [193]. Material‐level resilience may partly change this failure landscape. For example, a self‐healing soft robot driven by a two‐way shape‐memory‐alloy spring has demonstrated recovery of mechanical and robotic performance after damage, while an analytical dynamic model was developed to support robot design and optimization [194]. For hybrid‐intelligence architectures, this matters because the learned or adaptive component can be updated reliably only when relevant changes are observable or inferable from the available measurements. Sparse or poorly calibrated sensing may cause the model to attribute errors to the wrong source, while multimodal soft sensing can help separate deformation, contact, pressure dynamics, and environmental disturbance. Thus, sensing should be treated as part of the modeling architecture rather than as a separate instrumentation issue. Recent work on a multi‐material 3D‐printed thermoplastic polyurethane (TPU) soft pneumatic actuator further demonstrates how embedded sensing and deep learning can be combined for proprioceptive shape estimation, providing a direct link between compliant‐body sensing and data‐driven state reconstruction [195]. Soft manipulators are often only partially observable, because their internal deformation, stress distribution, and contact state cannot be measured directly. In adaptive MPC and online model‐learning studies [111, 159], sensing is not only used for feedback control but also for updating the model during deployment. Multimodal sensing, including pressure, flow, strain, force, tactile, vision, and motion‐tracking signals, can therefore provide the feedback needed to maintain model validity. In this setting, sensing and modeling become coupled: the model interprets incomplete measurements, while the measurements reveal when the model is no longer reliable. This interaction is especially important in tasks involving unknown objects, variable surfaces, human contact, or repeated manipulation over long operating periods.

Adaptive environmental learning can support several deployment‐relevant functions. Learning‐based force or position tracking [105] shows how model adaptation can improve interaction performance, while model‐based online learning for human‐wearable soft robots [196] demonstrates the relevance of adaptive control in human‐coupled soft robotic systems. In human‐coupled soft robotic systems, the robot, sensors, actuator structure, and human user form a coupled system whose effective dynamics can vary across users and operating conditions. More broadly, adaptive models can improve trajectory tracking when payloads change, adjust contact force regulation when surface stiffness varies, compensate for actuator drift or leakage, and maintain performance as material properties evolve over time. They can also support task transfer, where a model trained in one environment is updated efficiently for another without requiring full retraining. This is particularly valuable for soft robots, where collecting large amounts of high‐quality experimental data for every new task is often expensive and time‐consuming.

However, adaptation also introduces new challenges. Safe‐learning reviews in robotics [197] and safety‐critical control methods based on control barrier functions [198] show that online learning cannot be treated as a purely performance‐driven update process. Model‐driven approaches can provide stronger guarantees within predefined operating conditions but may generalize poorly, whereas data‐driven approaches can adapt to new contexts but often struggle to provide hard guarantees. For soft manipulators, the most useful direction is therefore a combined model–learning strategy, where adaptation improves performance while explicit boundaries constrain how the model and controller are allowed to change. Online model updates may improve short‐term performance while reducing stability, physical consistency, or interpretability. A model that adapts too aggressively may fit transient noise or contact disturbances rather than true system changes. For this reason, adaptive environmental learning should be combined with uncertainty estimation, physically constrained update rules, and safety‐aware control. The goal is not simply to make the model more flexible, but to make it flexible in a controlled and trustworthy way.

Overall, adaptive environmental learning represents a key step from modeling soft manipulators in the laboratory to deploying them in realistic environments. It shifts the role of the model from a fixed predictor to an evolving component of the robotic system, a direction reflected in recent online model‐learning work for soft robotics [111]. In future soft manipulation, the most useful models will not only predict deformation under known conditions, but also recognise when conditions have changed, update themselves using available sensory evidence, and remain compatible with safe and reliable control.

### 4.4 Trust, Safety, and Certification

As soft manipulators move from controlled laboratory demonstrations toward real‐world deployment, trust, safety, and certification become central modeling concerns. Safe‐learning research in robotics [197] and interpretable machine‐learning studies [199] make clear that a model that predicts accurately on a benchmark dataset is not necessarily suitable for safety‐critical or human‐facing operation. In soft robotics, this issue is especially important because the robot's body is compliant, its deformation is distributed, and its interaction with the environment is often difficult to describe using simple rigid‐body assumptions. The model must therefore support not only prediction and control, but also verification, uncertainty assessment, and safe decision‐making.

Trust in soft manipulator modeling has several dimensions. The first is physical credibility. A model should not only reproduce observed trajectories, but should also remain consistent with known mechanical behavior, such as force balance, geometric constraints, actuation limits, and dissipative response. Physics‐informed and hybrid modeling studies, including learned residual physics [102] and PINN‐based adaptive MPC with Cosserat rod structure [159], provide examples of how learned components can be coupled with physically meaningful structure. This is one reason why purely black‐box models can be difficult to justify in deployment contexts. Even when they give low prediction error, it may be unclear whether they will remain reliable under unseen contact conditions, payload changes, or long‐term material drift. Physics‐informed and hybrid models offer a more credible route because they retain explicit links to the underlying mechanics while still allowing data‐driven correction.

The second dimension is uncertainty. Soft manipulators often operate under partial observability, noisy sensing, and variable environments. A trustworthy model should therefore provide more than a single deterministic prediction. The distinction between different uncertainty types in Bayesian deep learning [200] is relevant here because deployment‐oriented soft robotic models need to indicate when predictions are uncertain, when the system has moved outside the training distribution, and when the controller should act conservatively. This is particularly important for contact‐rich tasks, where small errors in deformation or force prediction may lead to excessive contact force, unstable interaction, or task failure. Uncertainty‐aware modeling can support safer control by allowing the system to reduce speed, limit actuation, request human intervention, or switch to a more conservative control mode when confidence is low.

The third dimension is explainability. For scientific understanding, model interpretability helps reveal how actuation, deformation, loading, and environment interact. For deployment, explainable AI research [201] and interpretable machine‐learning theory [199] also suggest that explainability supports debugging, validation, and certification. Sparse models, reduced‐order dynamics, and physics‐informed residual structures can be easier to inspect than high‐dimensional black‐box predictors. This does not mean that every model must be fully analytical. Rather, the parts of the model that affect safety‐critical decisions should be understandable enough to support validation and failure analysis.

Safety also depends on how the model is embedded in the control architecture. Safe‐learning frameworks [197] suggest that a learned model may be acceptable for prediction or feedforward compensation, but less acceptable as the sole authority in a safety‐critical feedback loop. In many cases, a layered architecture is more appropriate. A nominal controller may use a learned or hybrid model for performance, while an independent safety layer enforces constraints on pressure, force, displacement, velocity, workspace, and contact state. Control‐barrier‐function‐based quadratic programmes [202] and learning‐based safety‐critical control [198] provide relevant mechanisms for this type of constraint enforcement. Such a structure allows data‐driven modeling to improve capability without removing the need for conservative physical limits.

Certification remains a difficult open problem for soft robotics. Existing collaborative robot standards, such as ISO/TS 15066 [203], and reviews of its use in hazard analysis and risk assessment [204], provide useful safety‐assurance principles. However, traditional certification frameworks are often based on predictable rigid‐body motion, well‐defined workspaces, and repeatable actuation. Soft manipulators challenge these assumptions because their shape, stiffness, and contact behavior can vary continuously. If adaptive or learning‐based models are used, the difficulty increases further, because the system may change its behavior after deployment. This suggests that certification of soft manipulators will require new forms of evidence, including model validation across operating regimes, uncertainty quantification, documented adaptation boundaries, fail‐safe mechanisms, and task‐specific safety metrics.

For future soft manipulation systems, trust and safety should therefore be considered from the beginning of the modeling process rather than added after the model has been trained. The choice of model structure, sensing modality, training data, uncertainty representation, and control interface all affect whether the system can be validated and certified. In this sense, trustworthy modeling is not separate from hybrid intelligence. It is one of its necessary conditions. A soft manipulator model becomes truly useful only when it is accurate enough to support action, interpretable enough to support understanding, adaptive enough to handle environmental change, and constrained enough to remain safe when its predictions are uncertain.

### 4.5 Case Examples in Soft Manipulation

The practical value of a soft‐manipulator model becomes clearer when it is connected to a real task. In many applications, the important question is not whether the model can reproduce every detail of the deformation field. A more useful question is whether the model helps the robot act better on hardware. This may mean smaller force error during contact, faster model construction before deployment, more stable manipulation with a compliant tool, or more accurate control in a constrained workspace. Recent studies provide useful examples of this shift. They show that modeling is becoming less of an isolated prediction problem and more of a task‐level support tool for control, adaptation, and deployment.

A first example is high‐precision control of a soft continuum manipulator for automated cable sorting. Gao et al. [205] proposed a hybrid offline–online data‐driven framework in which a multiscale neural network learns the manipulator model offline and multisensor feedback updates the model during operation. As shown in Figure 9A, the system uses a two‐segment cable‐driven soft continuum manipulator with integrated motion sensing and six‐cable actuation. The resulting controller achieved position deviations below 1 mm and orientation deviations below 0.8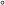, and was further demonstrated in multiwire cable sorting. This example illustrates how online model refinement can translate data‐driven modeling into precise task‐level control.

**FIGURE 9:**
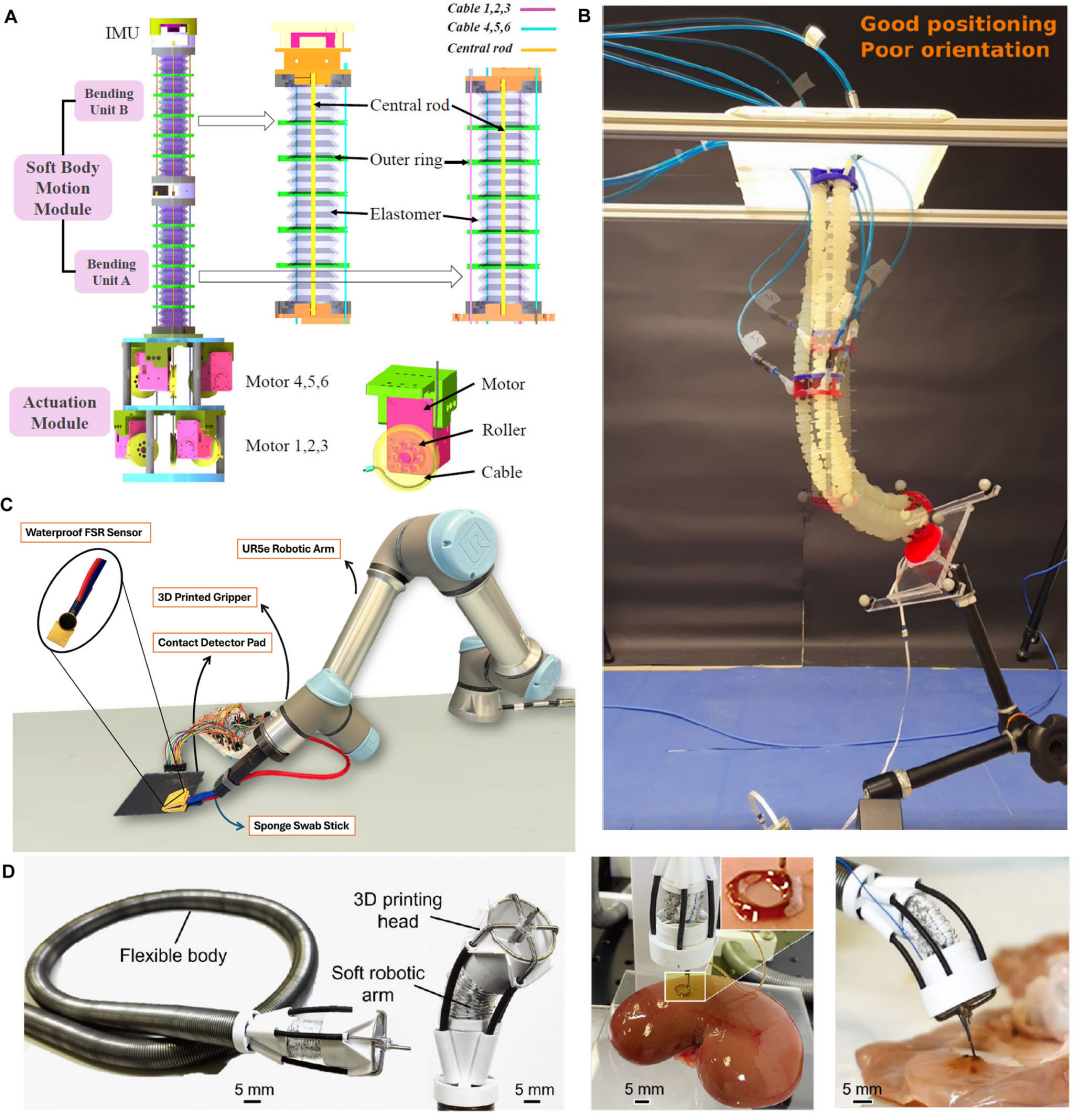
Application‐oriented evidence for model‐supported soft manipulation. (A) Two‐segment cable‐driven soft continuum manipulator used for high‐precision tracking and automated cable sorting, showing the soft‐body motion and actuation modules. Reproduced with permission under the terms of the Creative Commons CC BY license 4.0 [205]. Copyright 2024, Wiley‐VCH. (B) Sim‐to‐real soft‐arm pushing using model‐assisted deep reinforcement learning, illustrating a physical trial with accurate positioning but residual orientation error. Reproduced with permission under the terms of the Creative Commons CC BY license 4.0 [154]. Copyright 2024, Wiley‐VCH. (C) Robotic environmental swabbing, where a deformable swab tool is controlled through contact sensing and state‐adaptive Koopman control to improve surface coverage and force consistency. Reproduced with permission under the terms of the Creative Commons CC BY license 4.0 [76]. Copyright 2026, Wiley‐VCH. (D) Flexible in situ 3D bioprinting and endoscopic soft‐robotic system, showing the flexible manipulator, soft printing head, and representative ex vivo operation. Adapted with permission under the terms of the Creative Commons CC BY license 4.0 [206]. Copyright 2023, Wiley‐VCH.

A second example comes from model‐assisted learning for contact‐rich manipulation. Alessi et al. [154] used a dynamic Cosserat‐rod model to train a deep‐reinforcement‐learning policy for a three‐dimensional pushing task and transferred the controller to the physical soft arm using domain randomization. Figure 9B shows a representative physical pushing trial, in which accurate positioning is achieved while residual orientation error remains.

A related but more industrial example is robotic environmental swabbing for food safety. In this task, the compliant element is the swab tool rather than the manipulator body, but the control problem is still highly relevant to soft manipulation because the robot must regulate contact through a deformable interface. Mahmoudi et al. proposed a state‐adaptive Koopman LQR framework for a deformable swab tool [76]. As shown in Figure 9C, the system combines a robotic arm, a sponge swab stick, a contact detection pad, and a force‐sensitive sensing element. The task requires the robot to keep suitable contact pressure, maintain the tool angle, and cover the surface consistently. Their centroid‐based fuzzy force regulation reduced the average centroid error from 2.5 to 0.8 cm, increased the coverage percentage from 85% to 97%, and reduced the average force error from 10% to 3%. The task completion time increased from 83 to 95 s, which also shows a practical trade‐off: more stable contact and more complete coverage may come at the cost of a slower cycle. This is still useful evidence because deployment is usually judged by task reliability, not by speed alone.

Medical soft robots provide another example in which modeling is closely tied to deployment requirements. Thai et al. [206] developed a flexible in situ 3D bioprinting and endoscopic‐surgery system that combines a soft robotic arm, a flexible printing head, kinematic inversion, and learning‐based control. Figure 9D shows the flexible robotic system and representative ex vivo operation on porcine tissue. This example highlights the need for models that coordinate the deformation of the soft body with task‐specific end‐effector motion in confined medical environments.

Taken together, the examples in Figure 9 show how modeling supports deployment across distinct manipulation tasks. Dynamic task‐space modeling enables controlled surface interaction, model‐assisted deep reinforcement learning supports sim‐to‐real pushing, state‐adaptive Koopman control improves deformable‐tool swabbing, and model‐ and learning‐based control supports flexible in situ bioprinting and endoscopic operation. These examples reinforce that the appropriate modeling strategy depends on task dynamics, contact conditions, sensing, and computational requirements.

### 4.6 Evaluation Protocols for Hybrid‐Intelligence Architectures

If different modeling methods are combined within a hybrid‐intelligence architecture, they should not be evaluated only by prediction error. The main question is whether the overall system can support the intended robotic task under realistic operating conditions. For soft manipulators, this requires evaluation at several levels, including prediction, closed‐loop control, robustness, online updating, and safety [13, 16].

At the prediction level, the model should be assessed using outputs that are directly related to the task. These include end‐effector position, backbone shape, curvature, contact force, actuation pressure, or grasp success [207, 208, 209]. A weighted task error can be defined as [210]

$$E_{T}={\left({\hat{y}}_{T}-y_{T}\right)}^{T}W_{T}\left({\hat{y}}_{T}-y_{T}\right),$$

where $y_{T}$ is the measured task output, ${\hat{y}}_{T}$ is the predicted output, and $W_{T}$ gives more weight to variables that are more important for the task. This is useful because a model may have a small overall state error while still predicting the most important task variable poorly.

The model should also be tested inside the controller. Useful measures include tracking error, force‐control error, settling time, computation time, inference delay, and constraint violations. Recovery after disturbances, payload changes, or temporary sensing errors should also be considered [211]. A model that performs well in offline prediction may still be unsuitable for control if it is too slow or becomes unstable during online optimization [16, 71, 212].

Robustness should be tested by changing the conditions under which the model is used. For example, the material stiffness, damping, payload, actuation delay, contact condition, friction, and sensing noise can be varied within a realistic range [213]. The results should show both average performance and the worst cases. This helps determine whether the model works only near the calibration condition or remains useful when the robot or its environment changes. Methods such as domain randomization and dynamics randomization can be included in this evaluation [165, 166].

For architectures with online updating, the evaluation should also show whether the model remains aligned with the physical robot during operation. Relevant measures include state‐estimation error, parameter‐estimation error, convergence time, and the improvement obtained after updating. It is also useful to compare the results with the updating mechanism switched off. This comparison shows whether the system behaves as an adaptive digital twin or only as a fixed surrogate model [214, 215].

Safety and interpretability should be evaluated separately. The model should show which variables keep a physical meaning and where learned corrections are added. Possible safety measures include pressure‐limit violations, excessive contact force, workspace violations, unsafe control commands, and recovery after model or sensor failure [216]. For systems with uncertainty estimation or safety filters, the evaluation should also report how often the safety mechanism is activated, how much computation it requires, and whether it prevents constraint violations [217].

Ablation tests can be used to study the role of each part of the architecture [218]. For example, the physics model, learned residual, online update, uncertainty estimator, or safety filter can be removed one at a time. All versions should be tested using the same data and operating conditions. This makes it easier to see which components provide a real improvement.

Overall, the evaluation should consider accuracy, computation time, robustness, adaptation, and safety together. The aim is to show whether the hybrid architecture is suitable for the intended soft‐robot task, rather than whether it achieves the lowest prediction error on a single dataset.

## 5 Future Outlook: Toward Hybrid Intelligence

The future of soft manipulator modeling is unlikely to be defined by the emergence of a single dominant paradigm. Rather, progress will depend on the field's ability to move beyond method‐by‐method comparisons and toward integrated modeling frameworks that can support the full complexity of soft robotic systems across design, control, sensing, and deployment. In this sense, the next stage of the field is not about deciding whether continuum mechanics, sparse discovery, operator learning, neural dynamics, or physics‐informed learning is inherently superior. It is about understanding how these approaches can be positioned within a broader modeling architecture that remains physically meaningful, computationally efficient, and practically useful under real operating conditions. This shift is necessary because soft manipulators challenge many of the assumptions on which conventional robotic modeling has relied. Their behavior is shaped by distributed compliance, nonlinear deformation, material uncertainty, contact‐rich interaction, and strong dependence on both actuation and environment. As systems become more capable and application demands become more ambitious, modeling frameworks must do more than reproduce deformation under carefully controlled conditions. They must remain informative across changing tasks, uncertain environments, and different levels of required fidelity. This suggests that the field should increasingly think in terms of coordinated model ecosystems rather than isolated, standalone models.

In this Perspective, *hybrid intelligence* refers to a modeling architecture that coordinates physics‐based models, learned components, state estimation, adaptation, and task‐level decision‐making within explicit computational and safety constraints [30, 53, 197]. Compared with a conventional hybrid physics–machine learning model, it adds four operational mechanisms: role allocation between physics and learning, measurement synchronization, selective updating of uncertain states or parameters, and task‐ and safety‐aware use of the updated model [46, 102, 111, 159].

We therefore propose that a framework should be described as *hybrid intelligence* only when it retains explicit physical structure, assigns learning a defined operational function, updates its internal representation using sensory or task feedback, and uses the combined model to support decisions. These criteria distinguish an architecture‐level framework from a model in which physics and learning are combined only for offline prediction. For example, a physical model with a learned residual but without feedback, adaptation, or task‐level use is more accurately described as a hybrid physics–machine learning model rather than a complete hybrid‐intelligence framework [30, 102].

Within such an ecosystem, different representations may serve different purposes. High‐fidelity continuum and finite element formulations remain indispensable for mechanistic understanding, constitutive analysis, and design validation. Reduced‐order or structure‐preserving surrogates may be better suited to estimation, control, and fast prediction. Data‐driven corrections or learned latent representations may provide flexibility where purely analytical descriptions become impractical or incomplete. The value of *hybrid intelligence* lies precisely in this capacity to combine complementary model forms without collapsing them into a single undifferentiated framework. What matters is not whether one representation can do everything, but whether the overall modeling architecture can support the multiple functions required of a soft robotic system. An important implication is that future progress will likely depend on how effectively the field handles transitions across modeling scales and operating contexts. Soft manipulators are rarely used in only one regime. The same system may require detailed simulation during design, fast inference during operation, and updated representations when conditions shift or when the robot encounters previously unseen interactions. This places increasing importance on modeling strategies that can remain useful across these different contexts, either by enabling communication between representations or by supporting principled movement between levels of abstraction. In this sense, the future is about improving individual models and improving how models relate to one another across the robotic lifecycle.

A related challenge concerns robustness under variation. Soft manipulators are especially sensitive to fabrication differences, material ageing, drift, contact uncertainty, and environment‐dependent effects, all of which are difficult to capture completely in a fixed first‐principles description. This is one reason why learning‐based methods have become so attractive: they offer a route to absorbing behaviors that are only partially modeled, or that evolve over time. Yet the longer‐term opportunity is broader than simply attaching a correction term to a nominal physical model. The field is moving toward modeling frameworks that can remain useful even as the gap between nominal assumptions and operational reality widens. This will require greater emphasis on uncertainty, reliability, and the conditions under which a model can still be trusted. At the same time, models in soft robotics should increasingly be evaluated in terms of the roles they play within larger robotic systems. A model may act as a simulation tool, a control‐oriented surrogate, a virtual sensing mechanism, an inverse‐design engine, or a digital twin for monitoring system behavior over time. These roles differ substantially, and they place different demands on structure, speed, interpretability, and accuracy. As a result, future research may benefit from a fitness‐for‐purpose approach. A strong model for soft manipulators may not be the one that performs best across every metric, but the one best matched to the function it must serve within a broader intelligent system.

Under this definition, the main contribution of *hybrid intelligence* is architectural rather than tied to one specific modeling algorithm. It provides a structured way to coordinate models, sensing, estimation, adaptation, and action so that each component has a clear operational role [30, 46, 53]. Physics contributes mechanical structure, prior knowledge, and interpretable constraints, while learning provides residual correction, parameter adaptation, compression, and responsiveness to changing conditions [30, 102]. State estimation and online updating connect these components to the physical robot, and planning or control converts the updated model into task‐level action [111, 159]. This system‐level view also provides a direct link to the evaluation protocol in Section 4.6. The complete framework should be assessed through task accuracy, closed‐loop performance, robustness, online adaptation, and safety, rather than through prediction error alone [197].

Looking ahead, a central opportunity is to reconceptualise the soft manipulator model not as a single static entity, but as a component of a broader internal infrastructure for robotic intelligence. In this perspective, modeling is no longer treated solely as an offline descriptive exercise; instead, it functions as an active layer through which the robot integrates design knowledge, observed behavior, and operational requirements. Multiple representations may be invoked at different times, with their respective roles determined by fidelity needs, computational constraints, uncertainty, and task context. This shift moves the field beyond the traditional objective of identifying a single sufficiently accurate model, toward a more mature view in which intelligence arises from the principled coordination of multiple forms of representation. From this standpoint, the most important advances are unlikely to result from refining individual methods in isolation. Progress will depend on developing layered, context‐aware, and deployment‐oriented modeling frameworks. *Hybrid intelligence* provides a useful organising concept for this transition: it describes a shift from static model selection to integrated model utilization, and from methodological silos to coordinated representational strategies. For soft manipulators, such a reorientation may be essential for progressing from compelling experimental demonstrations to systems that are robust, versatile, and broadly useful in real‐world applications.

## 6 Conclusion

Soft manipulators bring robotics closer to the physical intelligence of living systems, but they also expose the limits of existing modeling methodologies. Their behavior is not governed by rigid kinematic chains alone, but by distributed deformation, material memory, actuation history, environmental contact and task‐dependent morphology. These properties make soft robots powerful, yet they also make them difficult to predict, control and trust. The central challenge is therefore no longer simply to build softer machines, but to build models that can make soft machines understandable and deployable.

This Perspective has traced an emerging transition from data‐driven discovery to hybrid intelligence. Sparse equation discovery offers interpretable reduced‐order dynamics; Koopman learning provides control‐compatible lifted representations; neural ordinary differential equations capture continuous‐time latent dynamics; physics‐informed neural networks constrain learning through mechanics; and hybrid physics machine learning models correct, adapt and extend imperfect physical descriptions. Each approach changes what a model can do, but none alone resolves the full complexity of soft manipulation.

The next advance will therefore come from modeling ecosystems rather than isolated algorithms. In this Perspective, *hybrid intelligence* refers to a modeling architecture that coordinates physics‐based models, learned components, state estimation, adaptation, and task‐level decision‐making within explicit computational and safety constraints. Its contribution lies in keeping physical structure visible, assigning learning to a defined source of uncertainty or computational cost, updating the model from measurements, and using the updated model to support action. A digital twin is one operational realization of this idea when the internal model is persistently synchronized with the physical robot and used for prediction or decision support. This definition provides a clearer basis for judging future work: a framework should improve task‐level capability while remaining physically credible, computationally usable, and safe within its stated operating range.

## Conflicts of Interest

The authors declare no conflicts of interest.

## Data Availability

The data supporting these findings are available upon request from the corresponding author.
